# Effects of long-term fluoride exposure are associated with oxidative biochemistry impairment and global proteomic modulation, but not genotoxicity, in parotid glands of mice

**DOI:** 10.1371/journal.pone.0261252

**Published:** 2022-01-27

**Authors:** Giza Hellen Nonato Miranda, Leidiane Alencar de Oliveira Lima, Leonardo Oliveira Bittencourt, Sávio Monteiro dos Santos, Michel Platini Caldas de Souza, Lygia Sega Nogueira, Edivaldo Herculano Corrêa de Oliveira, Marta Chagas Monteiro, Aline Dionizio, Aline Lima Leite, Juliano Pelim Pessan, Marília Afonso Rabelo Buzalaf, Rafael Rodrigues Lima

**Affiliations:** 1 Laboratory of Functional and Structural Biology, Institute of Biological Sciences, Federal University of Pará, Belém, PA, Brazil; 2 Laboratory of Clinical Immunology and Oxidative Stress, Pharmacy Faculty, Institute of Health Science, Federal University of Pará, Belém, PA, Brazil; 3 Evandro Chagas Institute, Ananindeua, PA, Brazil; 4 Department of Biological Sciences, Bauru Dental School, University of São Paulo, Bauru, SP, Brazil; 5 Department of Chemistry, University of Nebraska-Lincoln, Lincoln, Nebraska, United States of America; 6 Department of Preventive and Restorative Dentistry, School of Dentistry, São Paulo State University (UNESP), Araçatuba, SP, Brazil; Zagazig University, EGYPT

## Abstract

**Background:**

Fluoride has become widely used in dentistry because of its effectiveness in caries control. However, evidence indicates that excessive intake interferes with the metabolic processes of different tissues. Thus, this study aimed to investigate the effects of long-term exposure to F on the parotid salivary gland of mice, from the analysis of oxidative, proteomic and genotoxic parameters.

**Materials and methods:**

The animals received deionized water containing 0, 10 or 50 mg/L of F, as sodium fluoride, for 60 days. After, parotid glands were collected for analysis of oxidative biochemistry, global proteomic profile, genotoxicity assessment and histopathological analyses.

**Results:**

The results revealed that exposure to fluoride interfered in the biochemical homeostasis of the parotid gland, with increased levels of thiobarbituric acid reactive species and reduced glutathione in the exposed groups; as well as promoted alteration of the glandular proteomic profile in these groups, especially in structural proteins and proteins related to oxidative stress. However, genotoxic assessment demonstrated that exposure to fluoride did not interfere with DNA integrity in these concentrations and durations of exposure. Also, it was not observed histopathological alterations in parotid gland.

**Conclusions:**

Thus, our results suggest that long-term exposure to fluoride promoted modulation of the proteomic and biochemical profile in the parotid glands, without inducing damage to the genetic component. These findings reinforce the importance of rationalizing the use of fluorides to maximize their preventative effects while minimizing the environmental risks.

## 1. Introduction

The effectiveness, safety and cost-effectiveness of the use of fluoride (F) for caries control have been well-documented for several decades. Its widespread use around the globe comprises a variety of vehicles and modes of administration, including products for home use and professional application, in addition to community-based strategies [1]. However, overexposure to F can damage several biological processes, which can be associated with disorders in mitochondrial metabolism, induction of oxidative stress, interference with protein regulation and apoptotic process initialing [2].

The F interaction with different intracellular mechanisms triggers disturbances in both mineralized and soft tissues. Dental and skeletal fluorosis are well known clinical conditions caused by the exposure to high F levels, in a systemic manner [3]. However, the effects of excessive F intake on the metabolism of essential organs to the physiological balance of the oral environment (the salivary glands) have been investigated.

Responsible for 90% of the total saliva production [4], the major salivary glands consist of three pairs of glands known as parotid, submandibular, and sublingual. The saliva is a multifunctional fluid whose composition varies according to different physiological conditions. During mastication, the parotid gland is responsible for a large part of salivary production, mainly in the stimulated salivary secretion, with a flow twice as high as that of the submandibular gland [5].

The effects of F on the salivary glands have already been investigated by previous studies in animal models using low F concentrations. The results showed alterations in the glandular metabolism, such as increased accumulation of cyclic AMP in the parotid and submandibular glands [6], increased glycogen levels [7] and interference in carbohydrate metabolism in the submandibular glands [6, 8], in addition to stimulation of the amylase secretion in the parotid gland [6]. It is worth mentioning that several mechanisms are associated with F toxicity, as apoptotic pathways, oxidative stress and mitochondrial impairment [9–11].

Thus, these previous studies raise the need for studies on the toxicity of fluoride on glandular tissue. Therefore, this study proposes to investigate the effects of F on parotid salivary glands of mice exposed to 10 and 50 mg/L of F, through the evaluation of biochemical, proteomic, genotoxic and histopathological parameters.

## 2. Material and methods

### 2.1. Ethical aspects

The experimental protocol of this study was approved by the Ethics Committee on Animal Use (CEUA) from Federal University of Pará with protocol number 9469260117, following the NIH Guide for the Care and Use of Laboratory Animals.

### 2.2. Animals and treatment

Thirty male Swiss albino mice (21 days old) were acquired from the Federal University of Pará Animal House. The mice were housed in polypropylene cages and kept in an acclimatized room with a standard temperature and 12 hours light/dark cycles. Water and feed were available *ad libitum*.

After weaning, the animals were randomly divided into three groups of 10 animals. Two groups received deionized water containing 10 or 50 mg/L of F (as sodium fluoride, molecular weight = 41.99 from Sigma Chemical—USA) through voluntary consumption, over 60 days. The third, control group, received only deionized water for the same period. Dosages were established to simulate the long-term ingestion of F by humans at concentrations corresponding to approximately 2 and 10 mg/L of F [12–14]. The animals’ weight and volume of water ingested per cage were measured weekly.

### 2.3. Parotid gland collection procedures

**Fig 1:**
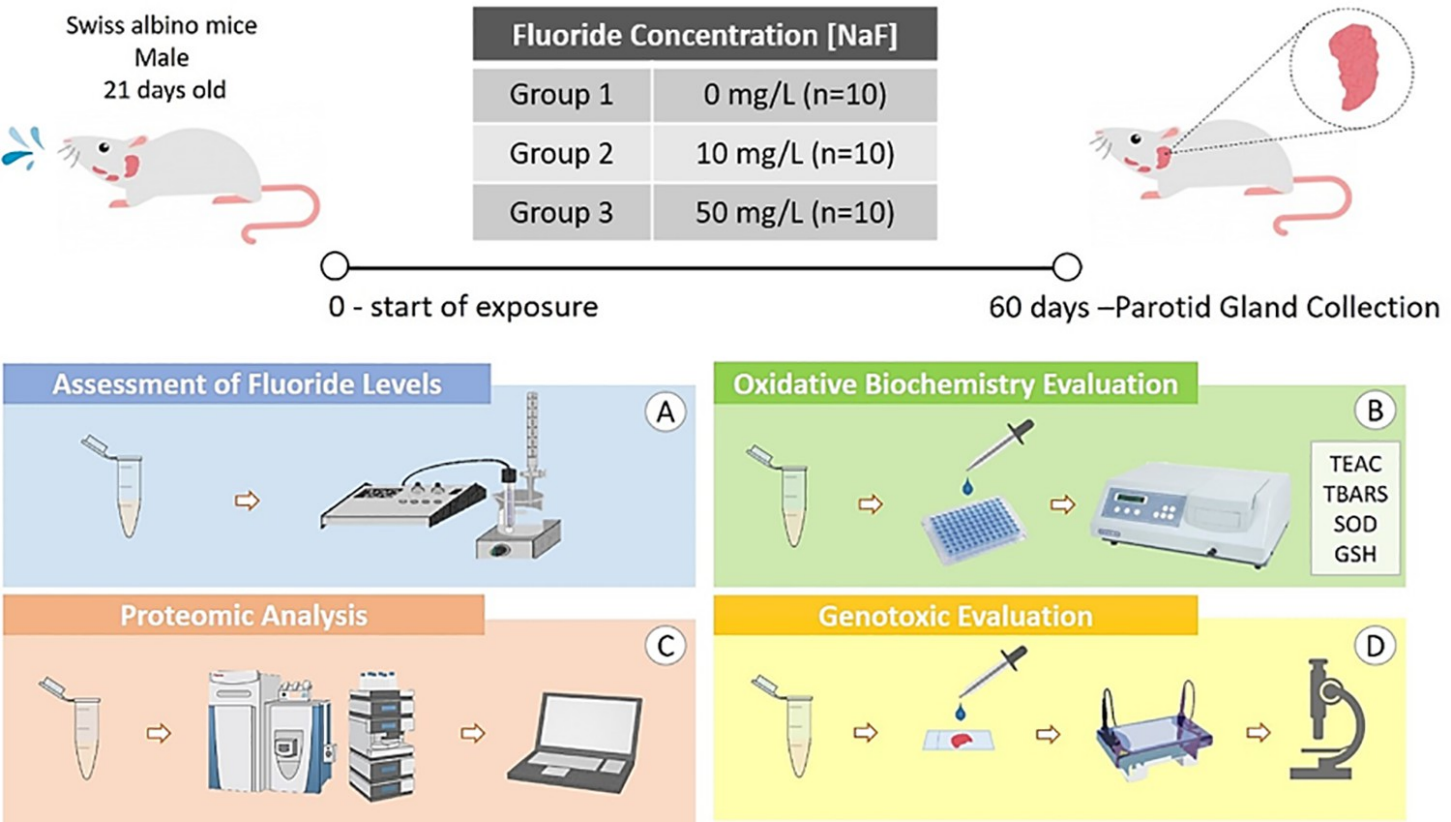
Methodological summary of the periods of experimentation, collection and analysis. 30 male Swiss albino mice (21 days old) received by voluntary consumption three fluoride (F) concentrations: 0 mg/L, 10 mg/L and 50 mg/L in deionized water (n = 10 per group). After 60 days of exposure, the animals were euthanized and the pair of parotid glands was collected for the following analyses: quantification of F concentration present in the glandular tissue (A); oxidative biochemistry (B), from Trolox Equivalent Antioxidant Capacity (TEAC), Thiobarbituric Acid Reactive Substances (TBARS), Superoxide Dismutase (SOD) and Reduced Glutathione (GSH) levels; analysis of protein expression profile (C), genotoxicity evaluation (D), based on the Comet Assay and histopathological analyses (F).

The animals were anesthetized with ketamine hydrochloride (90 mg/kg) and xylazine hydrochloride (10 mg/kg) and, after loss of reflexes they were euthanized. Then, surgery to collect the parotid salivary gland pair was performed, which was used for determination of F levels, oxidative biochemical, proteomic, genotoxic and histopathological analyses (Fig 1). The parotid gland pair of each animal was divided to all the analyses in such way the assays were performed in all the animals.

### 2.4. Assessment of F levels

For the F concentration analysis in the parotid gland, 0.2 g of glandular tissue to 0.5 mL of water were initially homogenized. Then F dosages in the tissues were performed as described by Taves [15] and, modified by Whitford [16], using a specific F^-^ electrode (Orion, model 9409) coupled to a reference calomel electrode (Accumet, # 13 -620-79), both coupled to a potentiometer (Orion model EA 940). The F concentrations were determined after acid-hexamethyldisiloxane (HMDS)-facilitated microdiffusion. The standard solutions used to perform the calibration curve (0.0048 to 0.19 μg F) were prepared in triplicate and diffused in the same manner as the samples. In addition, the non-diffused standards were prepared to have exactly the same concentrations of F^-^ as the diffused standards. The millivoltage readings (mV) were converted to μg F^-^ using Excel (Microsoft). A standard curve was adopted with a coefficient correlation of r 0.99. Results were expressed as μg/mL.

### 2.5. Oxidative biochemistry evaluation trials

To perform the analysis of oxidative parameters, the collected glandular tissue was washed with a saline solution and subjected to freezing with liquid nitrogen and then stored at—80° C. For the analyses, the samples were thawed and resuspended in 20 mM Tris-HCl, pH 7.4, at 4° C, by sonic disaggregation (approximate concentration 1 g/mL). The lysate was stored at—80° C until processing time.

#### 2.5.1. Determination of Trolox equivalent antioxidant capacity (TEAC)

The determination of the total antioxidant capacity was performed using the Trolox Equivalent Antioxidant Capacity (TEAC) technique. The 6-hydroxy-2,5,7,8-tetramethylchromono-2-carboxylic acid (Trolox, SigmaAldrich 23881–3) is a potent water-soluble antioxidant analog of vitamin E. The method proposed by Miller et al. [17] and modified by Re et al. [18], which is a colorimetric technique based on the reaction between 2,2-azinobis (3-ethylbenzothiazoline-6-sulfonic acid) (ABTS) (SigmaAldrich A1888) and potassium persulfate (K_2_S_2_O_8_; Sigma-Aldrich 60490), producing the radical cation ABTS^+•^ (2,2-azinobis 3-ethylbenzthiazoline-6-sulfonate), diammonium salt), and green / blue coloring chromophore [19]. The addition of antioxidants present in the sample to this preformed radical cation reduces it again to ABTS, on a scale dependent on the antioxidant capacity, antioxidant concentration and duration of the reaction. This can be measured by spectrophotometry by observing the change in absorbance read at 734 nm for 5 minutes. Results were expressed as μM/mL.

#### 2.5.2. Determination of reduced glutathione (GSH)

Determination of the concentrations of GSH was performed according to the method of Ellman [20]. This technique is based on the ability of GSH to reduce 5,5-dithiobis-2-nitrobenzoic acid (DTNB) (Sigma-Aldrich) to 5-thio-2-nitrobenzoic acid (TNB), which is quantified by spectrophotometry at a wavelength of 412 nm [19]. Samples were deproteinized with 2% trichloroacetic acid and the supernatant collected for analysis after centrifugation at 3000 rpm for 5 minutes. Initially, a 20 μL aliquot of each sample was taken and placed in a test tube containing 3 mL of PBS / EDTA buffer and 20 μL of distilled water to perform the first sample reading (T_0_), then 100 μL of DTNB and after 3 minutes the second sample reading (T_3_) was done. The difference in absorbance values (T_3_-T_0_) is proportional to the concentration of GSH, which was expressed in μM/mL.

#### 2.5.3. Determination of superoxide dismutase (SOD) activity

After preparation of the samples, a 50 μL aliquot was added to a buffer mixture, 0.075 mM cytochrome C, 1.5 mM hypoxanthine (Sigma-Aldrich) and 56 mM xanthine oxidase (Sigma-Aldrich, USA). The resulting solution was incubated at 37° C and protected from light and, after 15 minutes, a single reading in the 550 nm range was performed on a UV-1800 spectrophotometer. The absorbance values were applied in standard curve of cytochrome C to determine the enzymatic concentration, which was expressed in a unit of SOD/mg of protein, and a unit of SOD represents the amount of enzyme necessary to inhibit the rate of reduction of cytochrome C at pH 7.8 by 50%. The activity of the SOD enzyme was expressed in nMol/mL.

#### 2.5.4. Analysis of thiobarbituric acid reactive substances (TBARS)

The technical procedure of the method is the initial preparation of the potassium monobasic phosphate (KH_2_PO_4_ 75 mM, Synth, 35210) in acidified water (pH 2.5). This solution is used in the preparation of thiobarbituric acid (TBA) (10 nM). Then 100 μL of the sample was added to 500 μL of the 10 nM TBA solution. After, it was taken to the water bath (94°C for 60 minutes) and after incubation was allowed to cool to room temperature for 10 minutes. Then 2.0 mL of 1-butyl alcohol was added, vigorously homogenized in vortex and then subjected to centrifugation at 2500 rpm for 10 minutes; then 1.0 mL of the supernatant was collected for a spectrophotometric reading at 535 nm. The malondialdehyde (MDA) standard (1,1,3,3, tetrahydroxypropane—Sigma-Aldrich, T9889) was used to perform the standard curve and the results were expressed in μM/L.

### 2.6. Global proteomic analysis

#### 2.6.1. Extraction of proteins

The frozen salivary gland samples were homogenized. Then, protein extraction was performed by incubation in lysis buffer - 7M urea, 2M thiourea, 40 mM dithiothreitol (DTT), all diluted in an ammonium bicarbonate solution 50 mM (AMBIC) for 60 minutes under refrigeration and with continuous stirring. The samples were then centrifuged at 14,000 rpm for 30 minutes at 4° C for collection of the supernatant and thereafter, the protein content was measured in the samples pooled by the Bradford assay [21].

For 50 μL of each sample containing 50 μg of protein, 25 μL of 0.2% RapiGEST™ (Waters Co., Manchester, UK) was added and incubated at 37° C for 30 minutes. Then, 5 mM DTT was added to the solution and incubated at 37° C for 40 minutes. Subsequently, 10 mM iodoacetamide (IAA) was added and incubated for 30 minutes at room temperature under darkness. Protein digestion was performed with the addition of 10 μL trypsin (100 ng, Trypsin Gold Mass Spectrometry, Promega, Madison, USA) at 37° C overnight. Then, the samples were centrifuged at 14,000 rpm for 30 minutes at 6°C, the supernatants were collected and purified using C18 Spin columns (Pierce™). After purification, the samples were concentrated to an approximate concentration of 1 μg/μL and then, they were resuspended in 12 μL of ADH (1 pmol/μL) + 108μL of 3% acetonitrile and 0,1% formic acid for mass spectrometry analyses.

#### 2.6.2. Bioinformatics analysis

After protein extraction, peptide separation and identification were performed using a mass spectrometer coupled to the NanoAcquity UPLC-Xevo QTof MS system (Waters, Manchester, UK). The expression difference between the groups was obtained using the ProteinLynx Global Service software (PLGS, v 2.2.5, Waters) and the significance of the relative expression ratios was calculated using the Monte Carlo algorithm, considering p < 0.05 for down-regulated proteins with and 1-p > 0.95 for up-regulated proteins. The bioinformatics analysis was performed using ID numbers of the proteins from the Uniprot database to map their association with coded genes in the database. The software Cytoscape 3.0.4 (JAVA) was used to build networks of molecular interaction between the identified proteins, with the help of ClueGo applications.

#### 2.6.3. Over-representation analysis (ORA)

The ratio values were converted to log2 ratio, using the WPS spreadsheet editor. Then, cut-off values were applied for screening proteins with an expression value of 50% above or below in the exposed condition compared to the control. The analysis was performed considering only proteins with log2 ratio values of ≤ -0.58 or ≥ 0.58.

Using the Uniprot conversion tool, the protein codes were converted into the entrez ID record of the genes. The values -1 were assigned to proteins detected only in the control group and 1 to those presented exclusively in the sample exposed to the compound.

Using the R program and the Gene Set Enrichment Analysis package (EGSEA) [22] and using the list of entrez IDs and their respective log2 ratio values as input, an ORA analysis was performed. Gene Ontology BP (biological process), CC (cellular component) and MF (molecular function) databases were used in the analysis, as well as a set of molecular pathway genesets with information from different databases, such as REACTOME, Wikipathways, Panther, Netpath, HumanCyc and MsigDB. In the end, a list of overrepresented gene sets was generated. A p-value ≤ 0.05 was considered significant.

### 2.7. Genotoxic evaluation

The comet assay was made according to adaptations of the method of Zhang et al. [23]. The glandular tissue, after dissection, was immersed in DMEM (Dulbecco’s modified Eagle’s medium) solution with collagenase and maintained in the incubator at 37° C for 90 minutes. Then, the tissue was filtered and the resuspended tissue was centrifuged for 5 minutes at 200 rpm. The pool of cells formed was used for the assay.

For each slide, an aliquot of 20 μL of cell suspension and 120 μL of low melting point agarose was prepared. The aliquot content was applied to the slides previously prepared with 1.5% normal melting point agarose and coverslips were placed thereon; the material was incubated at 4° C for 20 minutes. Thereafter, the coverslips were removed and the slides were dipped in a lysis solution and incubated in the refrigerator for 12–78 h hours.

After incubation, the slides were placed in an electrophoresis cell and covered with cold electrophoresis solution for 20 minutes to initiate the run (electrophoresis) at 300 mA and 20V for 20 minutes. The slides were then overlaid with neutralizing solution three times and, after drying, were immersed in 100% ethanol for 10 minutes. After drying again, 15 μL of DAPI/Antifade solution was added and the coverslips were replaced.

After the slides were prepared, the comet assay determination was performed by capturing the images using a fluorescence microscope (Leica Microsystems, Wetzlar, Germany, with a magnification of 400x) connected to a CCD camera (charge-coupled device). The images were analyzed using Comet Assay IV^TM^ software (Perceptive Instruments). For each animal, two slides were photographed, recording 50 cells in each. Considering that cells with damaged DNA have a greater migration of chromosomal fragments outside the nucleus [24], the parameter used to determine DNA damage was the percentage of DNA in the tail.

### 2.8. Histopathological analyses

The glands were immersed in 10% formalin for 24 hours and then dehydrated in solutions of increasing concentration of ethanol, clarified in xylol and embedded in Paraplast (Mccormick Scientific, USA). Semi-serial sections of the entire extension of the glands were obtained at 6 micrometers thick and stained with Hematoxylin and Eosin. The sections were qualitatively inspected for their secretory units and ductal system, in addition to the surrounding stroma, for possible changes in the glandular epithelium, lining epithelium and connective tissue itself. The representative photomicrographs were taken by a DS-Fi3 microscope camera attached to the Nikon Eclipse Ci H550s brightfield microscope

### 2.9. Statistical analysis

For analysis of the data, the normality of each group was initially tested using the Kolmogorov-Smirnov method. Data on F concentration, oxidative biochemistry and genotoxic evaluation were analyzed by means of one—way analysis of variance (ANOVA), followed by the Tukey test. The statistical design was performed using Graphpad 5.0 software, considering a significance value of p < 0.05. The results were represented as mean and standard deviations.

The proteomic data global analysis was performed using the ProteinLynx Global Service software (PLGS, v 2.2.5, Waters), and the significance of the relative expression ratio was calculated using the Monte-Carlo algorithm, considering p < 0.05 for down regulated proteins and 1-p > 0.95 for up regulated proteins.

The proteomic data global analysis was performed using the ProteinLynx Global Service software (PLGS, v 2.2.5, Waters), and the significance of the relative expression ratios was calculated using the Monte-Carlo algorithm, considering p < 0.05 for down regulated proteins and 1-p > 0.95 for up regulated proteins.

## 3. Results

### 3.1. The F levels in the parotid glands were significantly increased only in the 50 mg/L of F group

**Fig 2:**
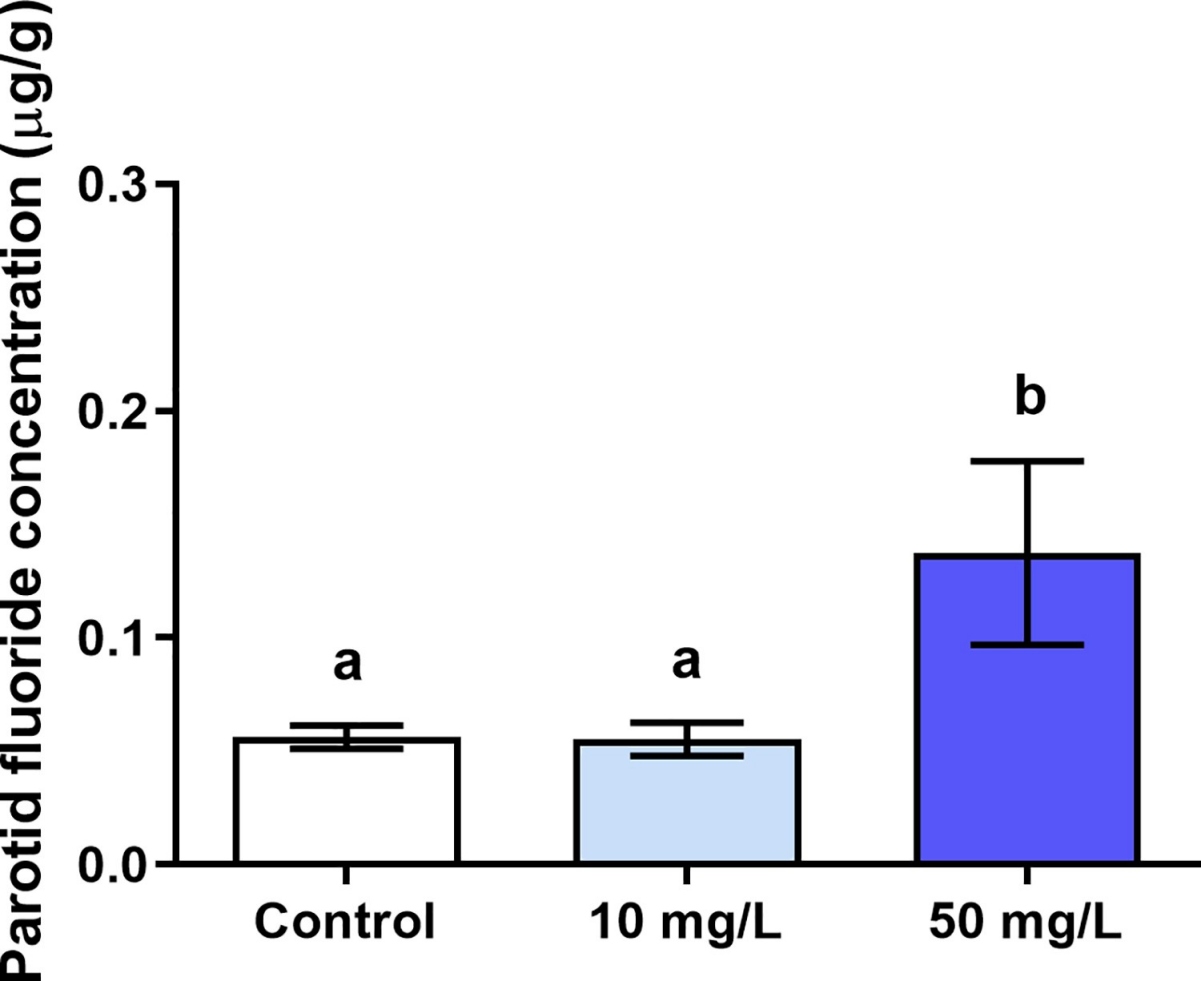
Fluoride concentration in the parotid glands. Analysis of fluoride (F) concentration in the parotid glands of mice (n = 30) in μg/g, after 60 days of exposure to 0 mg/L, 10 mg/L and 50 mg/L of F (wet weight). Results are expressed as mean ± standard deviations. The different letters on the columns indicate significant differences among the groups according to One-way ANOVA test, with Tukey posttest, p< 0.05.

The F levels present in the parotid glands of the group that received 50 mg/L of F treatment (0.14 ± 0.070 μg/g) were significantly higher than the control group (0.06 ± 0.01 μg/g; p = 0.03) and 10 mg/L of F (0.06 ± 0.02 μg/g). There was no statistical difference between the 10 mg/L group and the control group (p>0.05) (Fig 2).

### 3.2. The F exposure increased the concentration of TBARS and GSH levels in the 10 mg/L and 50 mg/L of F groups

**Fig 3:**
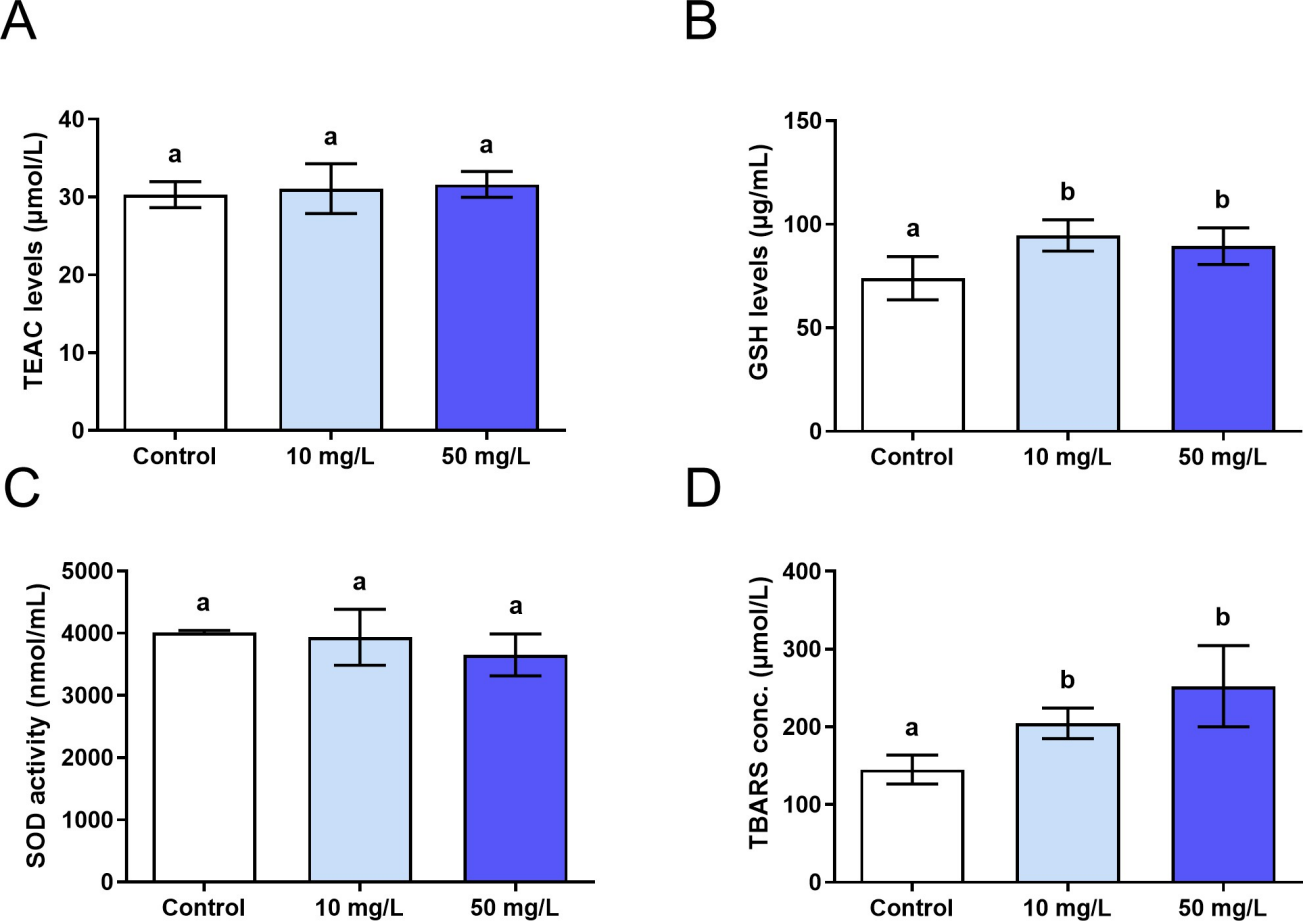
Oxidative biochemistry assessment. Analysis of oxidative biochemistry in the parotid glands of mice (n = 30, 10 per group) exposed for 60 days to 0 mg/L, 10 mg/L and 50 mg/L of fluoride (F). Results are expressed as mean ± standard deviation of the values referring to the oxidative parameters: A) TEAC levels; B) GSH levels; C) SOD activity; D) TBARS concentration. The different letters on the columns indicate significant differences among the groups according to One-way ANOVA test, with Tukey posttest, p< 0.05.

The analysis of oxidative biochemistry showed a significant increase in the TBARS concentration (p< 0.00) and GSH content (p = 0.00) in the exposed groups (10 mg/L and 50 mg/L of F) when compared to the control. However, the levels of TEAC (p = 0.44) did not present statistical differences between the groups, as well as the SOD activity (p = 0.23). No analyzed biochemical parameters revealed statistical differences between the 10 mg/L and 50 mg/L groups (Fig 3).

### 3.3. The exposure to F altered the protein expressions associated with different biological processes

The number of proteins with differences in expression or expressed uniquely varied between comparisons 10 mg/L of F vs. control and 50 mg/L of F vs. control. In the first comparison, the number of proteins upregulated in the exposed group were 68 and, in the down-regulated were 132; the proteins only expressed in 10 mg/L group were 279 and those expressed solely in control were 222 (S1 Table). In the comparison 50 mg/L of F vs. control, the upregulated proteins in the exposed group totaled 19, while downregulated totaled 138; those expressed exclusively in the 50 mg/L group were 190 and 238 in the control group (S2 Table).

**Fig 4:**
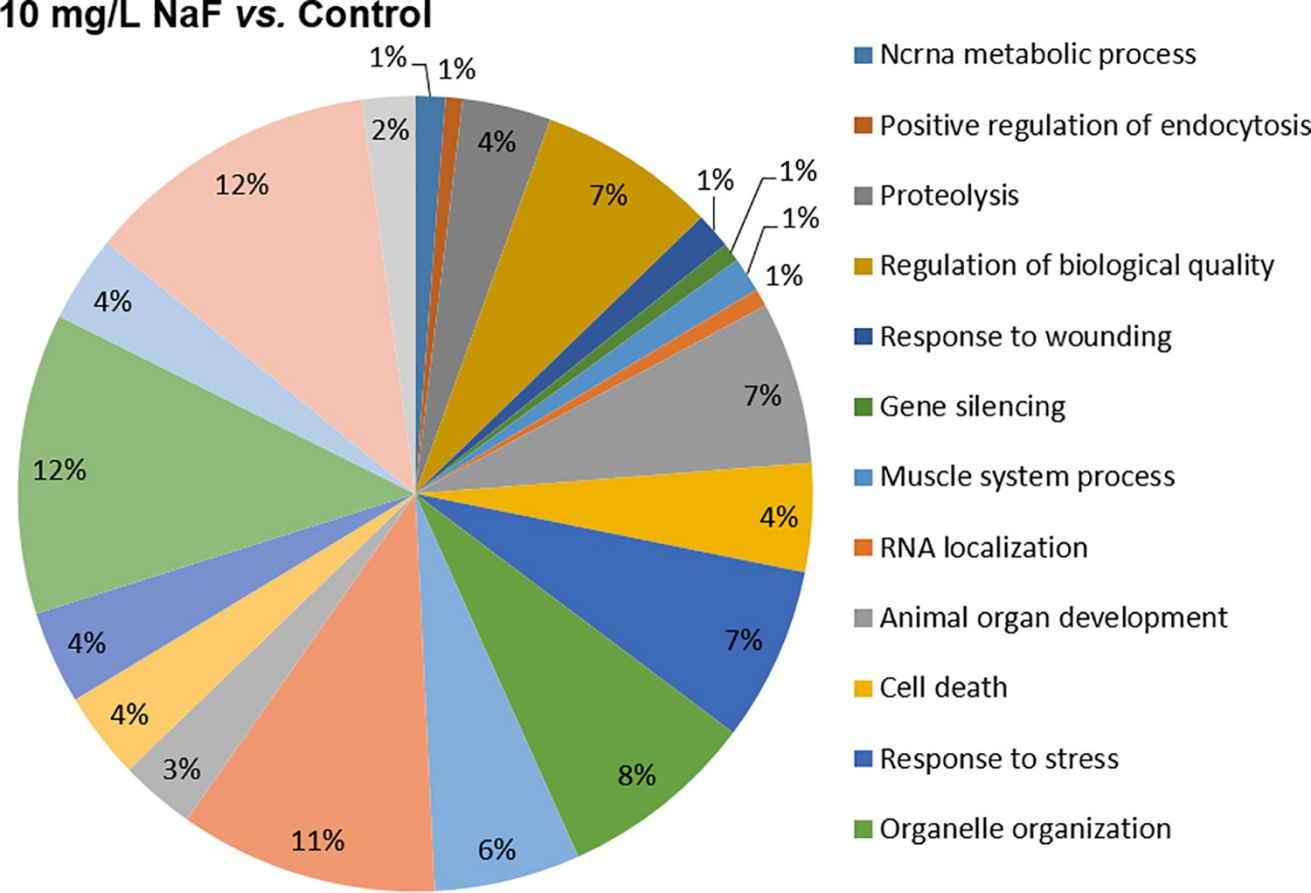
Functional distribution of proteins (10 mg/L of F vs. control group). Functional distribution of proteins identified with different expressions in the parotid glands of mice (n = 30, 10 per group) exposed for 60 days to 10 mg/L *vs*. 0 mg/L of F. Categories of proteins based on gene ontology (GO) selected for biological processes. Significant terms (Kappa = 0.4) and distribution according to percentage of number of gene associations. The protein accession number was provided by the Uniprot database. The GO was evaluated according to Cytoscape® software 3.4.0, using the ClueGo® plugin.

**Fig 5:**
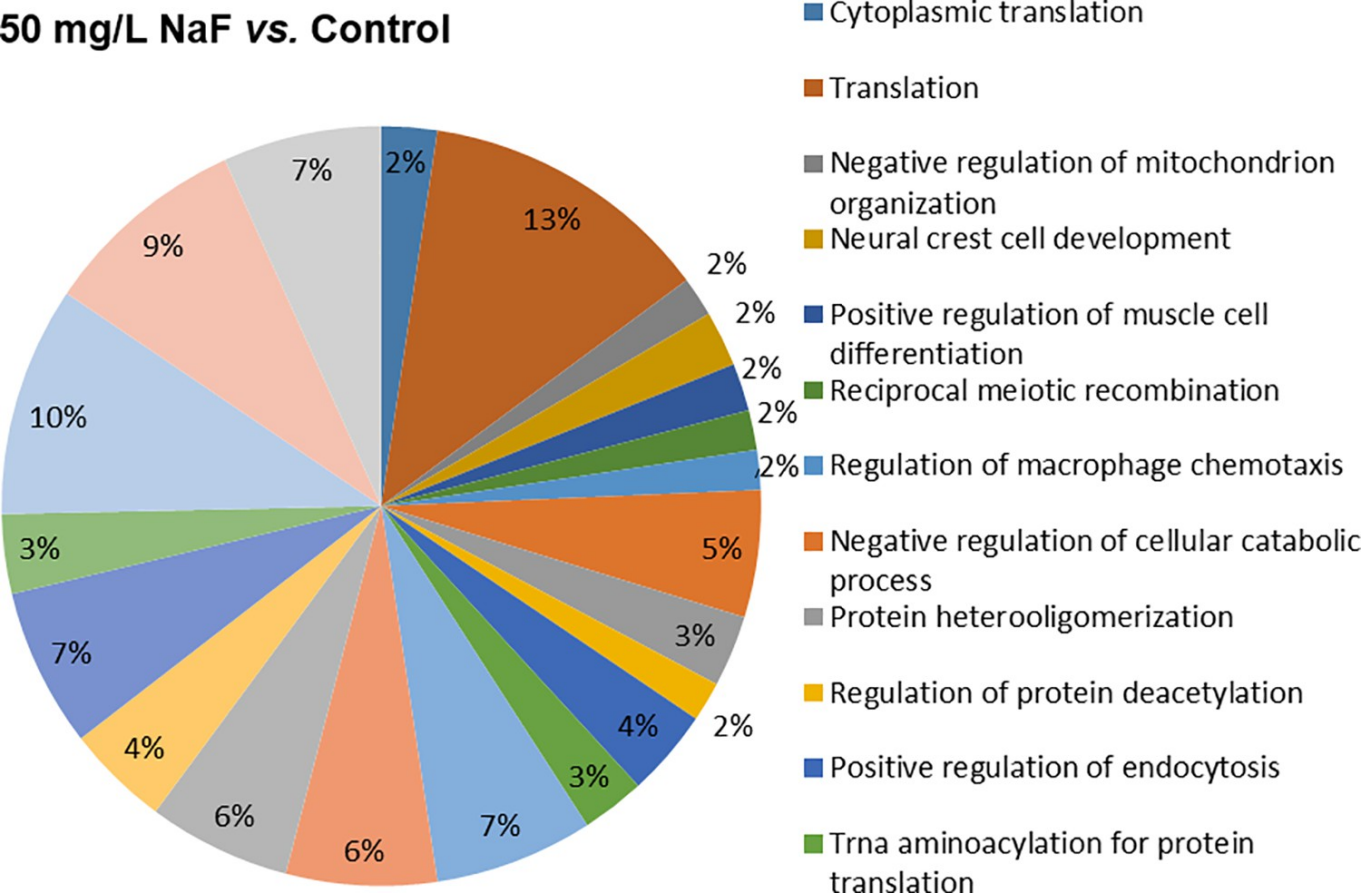
Functional distribution of proteins (50 mg/L of F vs. control group). Functional distribution of proteins identified with different expressions in the parotid glands of mice (n = 30, 10 per group) exposed for 60 days to 50 mg/L *vs*. 0 mg /L of F. Categories of proteins based on gene ontology (GO) selected for biological processes. Significant terms (Kappa = 0.4) and distribution according to percentage of number of gene associations. The protein accession number was provided by the Uniprot database. The GO was evaluated according to Cytoscape® software 3.4.0, using the ClueGo® plugin.

Regarding the evaluation of the functional groups of proteins, based on the biological process, the group exposed to 10 mg/L of F presented alterations in 21 functional categories. The categories with the highest percentage of associated genes were: *Organonitrogen compound metabolic process* (12.2%), *Cellular component organization or biogenesis* (12%), *Anatomical structure development* (10.5%), *Organelle organization* (8%), *Regulation of biological quality* (7.3%), *Response to stress* (7.1%) and *Animal organ development* (6.6%). The 50 mg/L of F group also showed 21 affected functional categories, and the biological processes with the highest percentage of associated genes were: *Translation* (12.5%), *Regulation of cell morphogenesis* (9.8%), *Actin filament organization* (8.8%), *Regulation of cell morphogenesis involved in differentiation* (6.8%), *ATP metabolic process* (6.8%) and *Regulation of actin cytoskeleton organization* (6.8%). Figs 4 and 5 represent the functional classification according to the biological process for the comparison of the 10 mg/L of F vs. control and 50 mg/L of F vs. control, respectively.

**Fig 6:**
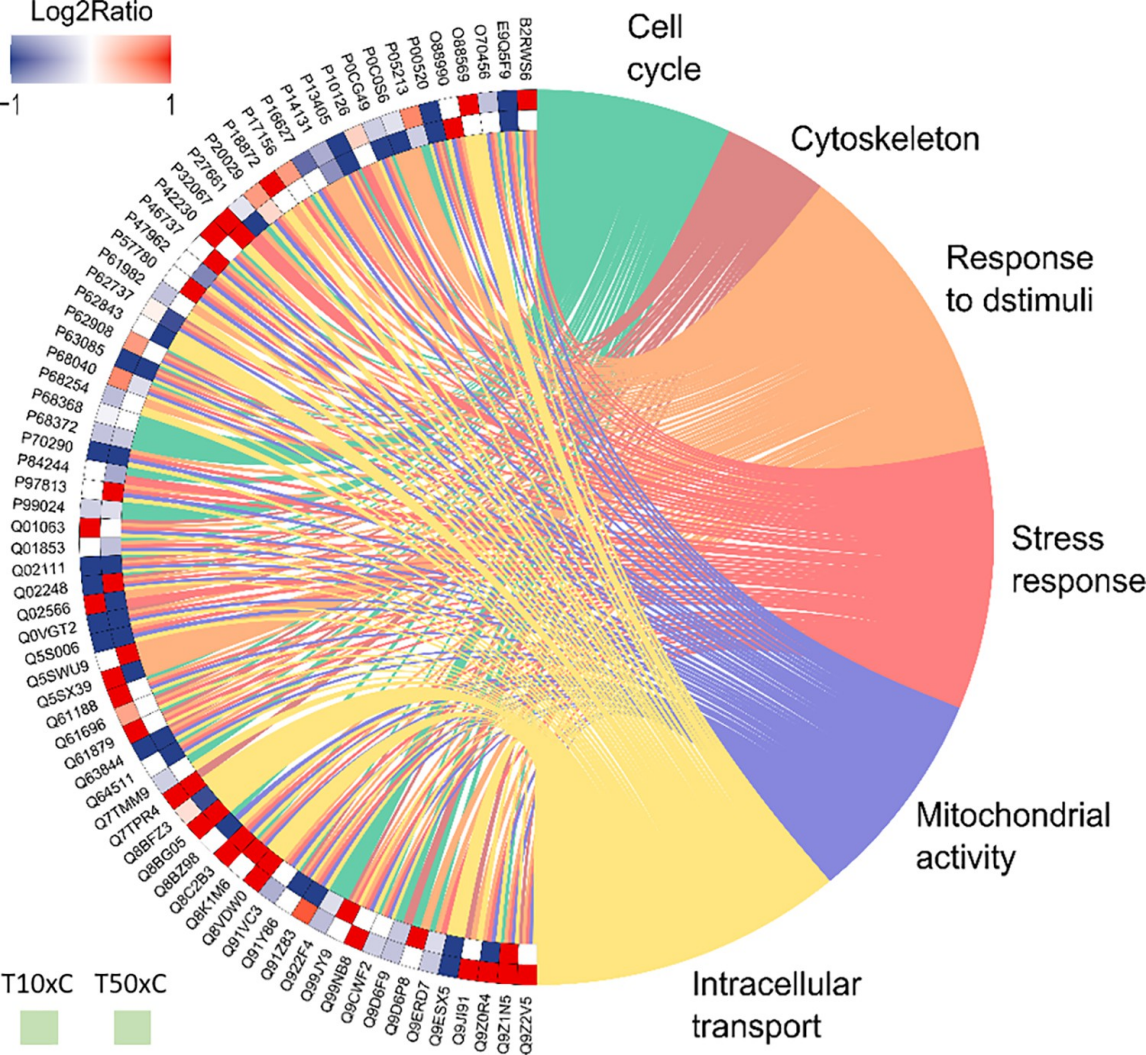
Enrichment analysis. Over-represented proteins in the parotid glands of mice (n = 30, 10 per group) exposed for 60 days to F concentrations: 10 mg/L of F *vs*. control (T10xC) and 50 mg/L of F *vs*. control (T50xC). Protein categories based on the gene ontology (GO) selected for biological processes, cellular component and molecular function. The external and internal protein sequences represent the comparisons between T10xC and T50xC, respectively. The color indicates the differential expression of each protein, that is represented with its access code. Red indicates overexpression and blue, down-regulation. The tonality varies according to the intensity of down or up regulation status. According to the Uniprot database, the access numbers of the proteins over and under-represented correspond to: *Histone acetyltransferase p300* (B2RWS6), *Histone-lysine N-methyltransferase SETD2* (E9Q5F9), *14-3-3 protein sigma* (O70456), *Heterogeneous nuclear ribonucleoproteins A2/B1* (O88569), *Alpha-actinin-3* (O88990), *Tyrosine-protein kinase ABL1* (P00520), *Tubulin alpha-1B chain* (P05213), *Histone H2A*.*Z* (P0C0S6), *Polyubiquitin-B* (P0CG49), *Elongation factor 1-alpha 1* (P10126), *Retinoblastoma-associated protein* (P13405), *40S ribosomal protein S16* (P14131), *Heat shock 70 kDa protein 1-like* (P16627), *Heat shock-related 70 kDa protein 2* (P17156), *Guanine nucleotide-binding protein G(o) subunit alpha* (P18872), *Endoplasmic reticulum chaperone BiP* (P20029), *Histone* (H2AX P27661), *Lupus La protein homolog* (P32067), *Signal transducer and activator of transcription 5A* (P42230), *Lys-63-specific deubiquitinase BRCC36* (P46737), *60S ribosomal protein L5* (P47962), *Alpha-actinin-4* (P57780), *14-3-3 protein gamma* (P61982), *Actin*, *aortic smooth muscle* (P62737), *40S ribosomal protein S15* (P62843), *40S ribosomal protein S3* (P62908), *Mitogen-activated protein kinase 1* (P63085), *Receptor of activated protein C kinase 1* (P68040), *14-3-3 protein theta* (P68254), *Tubulin alpha-4A chain* (P68368), *Tubulin beta-4B chain* (P68372), *55 kDa erythrocyte membrane protein* (P70290), *Histone H3*.*3* (P84244), *Phospholipase D2* (P97813), *Tubulin beta-5 chain* (P99024), *cAMP-specific 3’*,*5’-cyclic phosphodiesterase 4D* (Q01063), *Transitional endoplasmic reticulum ATPase* (Q01853), *Protein kinase C theta type* (Q02111), *Catenin beta-1* (Q02248), *Myosin-6* (Q02566), *Zinc finger protein GLI2* (Q0VGT2), *Leucine-rich repeat serine/threonine-protein kinase 2* (Q5S006), *Acetyl-CoA carboxylase 1* (Q5SWU9), *Myosin-4* (Q5SX39), *Histone-lysine N-methyltransferase EZH2* (Q61188), *Heat shock 70 kDa protein 1A* (Q61696), *Myosin-10* (Q61879), *Mitogen-activated protein kinase 3* (Q63844), *DNA topoisomerase 2-beta* (Q64511), *Tubulin beta-2A chain* (Q7TMM9), *Alpha-actinin-1* (Q7TPR4), *Beta-actin-like protein 2* (Q8BFZ3), *Heterogeneous nuclear ribonucleoprotein A3* (Q8BG05), *Dynamin-3* (Q8BZ98), *Histone deacetylase 7* (Q8C2B3), *Dynamin-1-like protein* (Q8K1M6), *ATP-dependent RNA helicase DDX39A* (Q8VDW0*)*, *Eukaryotic initiation factor 4A-III* (Q91VC3), *Mitogen-activated protein kinase 8* (Q91Y86), *Myosin-7* (Q91Z83), *Tubulin beta-6 chain* (Q922F4), *Actin-related protein 3* (Q99JY9), *Ubiquilin-4* (Q99NB8), *Tubulin beta-2B chain* (Q9CWF2), *Tubulin beta-4A chain* (Q9D6F9), *Calmodulin-like protein 3* (Q9D6P8), *Tubulin beta-3 chain* (Q9ERD7), *H/ACA ribonucleoprotein complex subunit DKC1* (Q9ESX5), *Alpha-actinin-2* (Q9JI91), *Intersectin-1* (Q9Z0R4), *Spliceosome RNA helicase Ddx39b* (Q9Z1N5), and *Histone deacetylase 6* (Q9Z2V5).

The ORA analysis identified, from the functional enrichment, proteins with differences in expression in the two exposed groups. In the 10 mg/L of F vs. control comparison, the number of proteins upregulated in the exposed group were 29, and downregulated were 28. In the comparison 50 mg/L of F vs. control, the upregulated proteins in the exposed group totaled 16, while downregulated totaled 34 (S3 Table). Furthermore, it was possible to observe in both the exposed groups that the over and under expressed proteins were related to different biological processes, such as cell cycle, cytoskeleton, response to stimuli, stress response, mitochondrial activity, and intracellular transport. Fig 6 represents the functional enrichment analysis.

### 3.4. Exposure to F had no genotoxic effect on DNA integrity

**Fig 7:**
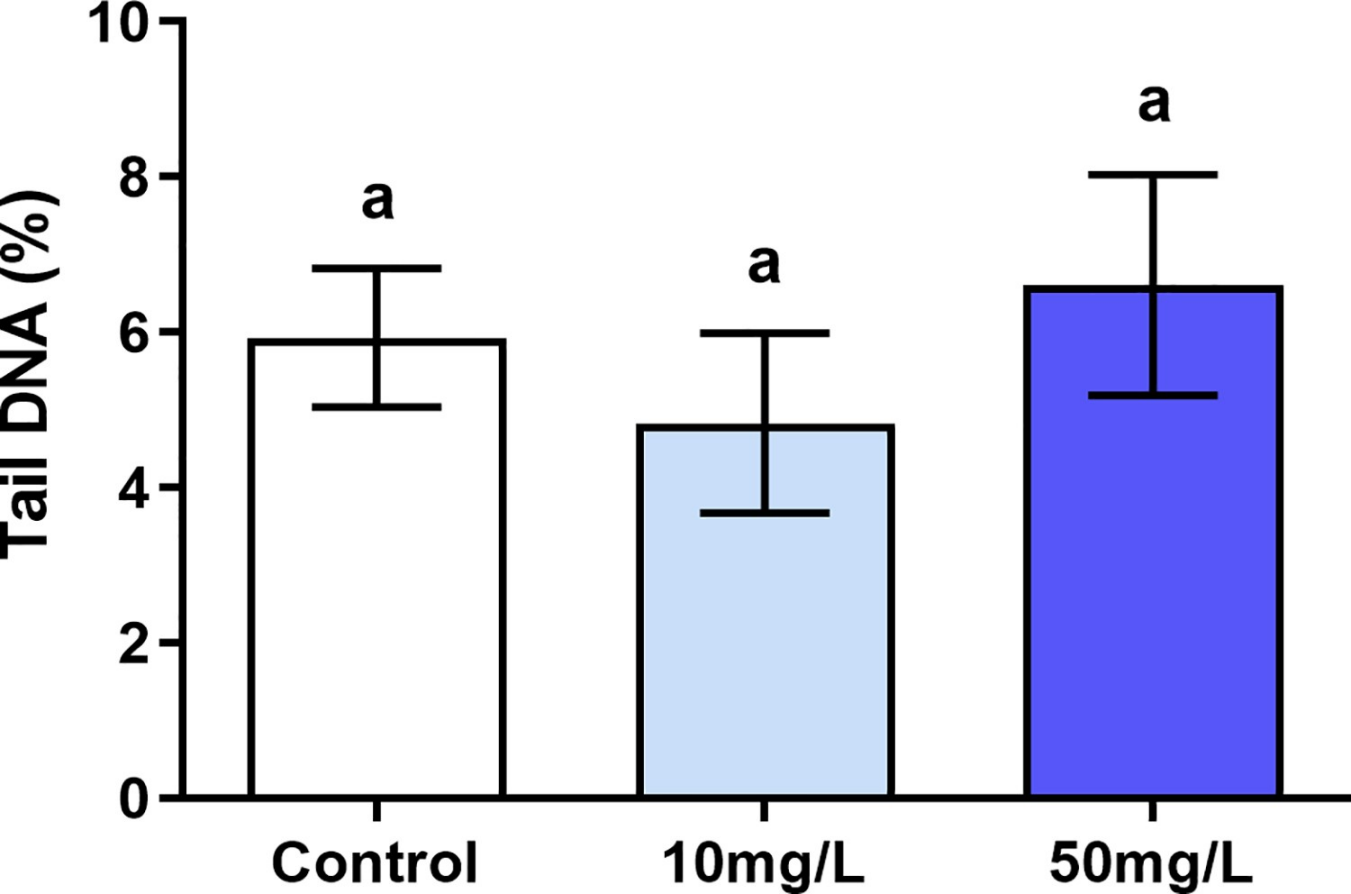
Genotoxicity assessment. Evaluation of genotoxicity in the parotid glands of mice (n = 30, 10 per group) exposed for 60 days to F concentrations: 0 mg/L, 10 mg/L and 50 mg/L. The genotoxic damage was represented as a percentage of DNA in the tail and expressed as a mean ± standard deviation. The letters on the columns indicate significant differences among the groups according to One-way ANOVA test, with Tukey posttest, p< 0.05.

The genotoxic analysis showed no statistical difference (p = 0.06) in relation to the estimate of DNA damage (% of DNA in the tail) between 10 mg/L of F (4.83 ± 1.16), 50 mg/L of F (6.61 ± 1.42) and control groups (5.93 ± 0.89) (Fig 7).

### 3.5. No histopathological were diagnosed

**Fig 8:**
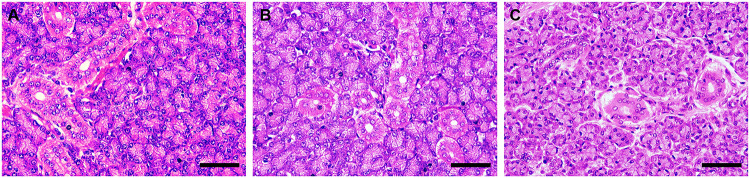
Histopathological analysis. Evaluation of morphological changes in the parotid glands of mice (n = 30, 10 per group) exposed for 60 days to F concentrations: 0 mg/L, 10 mg/L and 50 mg/L. Photomicrographs of sections stained in Hematoxylin and Eosin. A 0 mg/L, B 10 mg/L and C 50 mg/L. Scale bar: 50 μm.

Histopathological inspection did not show changes in the acinar, ductal or stroma organization, and no changes in proportions, size and color intensity between groups were evidenced (Fig 8).

## 4. Discussion

The results of the present study demonstrated that the long-term F administration for 60 days in mice was able to alter the proteomic profile, as well to modulate oxidative biochemistry in the parotid glands, however, without damaging the DNA of the cells that make up the glandular tissue nor in the tissue organization of these glands.

The exposure model proposed in this investigation was used to simulate chronic F ingestion by humans at concentrations that correspond to approximately 2 and 10 mg/L of F [13, 14]. In order to establish comparable serum F levels, evidence suggests that the F content in drinking water of rodents should exceed four to five times the concentration for humans [14, 25]. Thus, the literature presents several animal model studies that used similar doses to estimate the effect of F on tissues such as liver, kidney [11], intestine [13], nervous tissue [26] and blood [27].

Long-term F exposure of mice to concentrations of 10 and 50 mg/L was previously assessed by our study group in blood samples. The findings indicated that there was an increase of plasma F levels in both the lowest and highest doses [12]. In contrast, the present study, evaluating the parotid glands, showed that only in the group that received the highest dose was there an increase in the F concentration. This suggests that although there was no accumulation of F in the glandular tissues at 10 mg/L of F, the F concentration in the blood was higher. Thereby, a greater amount of F would be available to interact with the tissue structure of the parotid glands in these animals when compared to the control group.

Furthermore, the previous study [12] also demonstrated that F intake was able to alter parameters related to oxidative stress in blood in the two proposed concentrations. Similarly, parotid gland analysis indicated changes in oxidative biochemistry in the exposed groups. This is consistent with findings in submandibular glands of rats exposed to 15 mg/L of F [28].

The oxidative stress condition is established when there is an imbalance between the production of pro-oxidants and antioxidant agents. This event can be triggered by an increase in the production of free radicals in the intracellular environment and/or a decrease in the effectiveness of the defense system [29]. F has been widely reported as an inducer of oxidative stress that is due to the ability to modulate the intracellular redox state. Evidence suggests that F may impair mitochondrial function by decreasing cellular respiration and ATP production [3]. Since mitochondria are the main source of reactive oxygen species (ROS) production, F toxicity would be associated with ROS induction and reduction of cellular antioxidant defenses against oxidative damage [3].

In order to prevent damage from the oxidation of cellular components, antioxidant defense mechanisms have been developed by the body to neutralize the action of reactive species and prevent the compromise of important biological functions [30]. In the present study, three antioxidant parameters were analyzed including the TEAC levels, the SOD activity and the GSH content, in addition to a pro-oxidant factor represented by TBARS, in order to evaluate the cellular redox state through the tissue interaction with the levels of F administered.

The TEAC is an assay that quantifies the antioxidant activity based on the altered ability of biological systems to modulate the concentration of reactive species [29, 31]. In this study, the antioxidant potential measured by this assay showed no difference between the exposed and control groups. However, it is worthy to highlight that F can also act on other stress pathways mediated by enzymatic activity.

SOD is part of the enzymatic antioxidant system and it is responsible for catalyzing the conversion of superoxide radical (O_2_-) to hydrogen peroxide (H_2_O_2_) [32]. Our findings showed that the SOD activity was not altered by F, which is consistent with previous studies of the parotid gland [28]. F can act as an inhibiting agent of SOD activity due to a binding of F^-^ ion to the active site of the enzyme. However, the degree affinity of F^-^ for the active site may vary according to the source of SOD [33]. The F^-^ may have more than twice the affinity for SOD isolated from yeast than from the bovine liver [34]. Therefore, it is possible that the unaltered SOD activity in the parotid gland is related to this mechanism.

GSH, in turn, participates in the neutralization of the radical hydrogen peroxide (H_2_O_2_), which occurs at the expense of converting GSH into oxidized glutathione (GSSG) [29]. In this study there was an increase in the content of GSH in both exposed groups. This suggests that F triggered protection mechanisms by increasing antioxidant factors in an attempt to protect the integrity of cell biomolecules.

The results of this study also showed an increase in TBARS concentration in both the 10 mg/L and 50 mg/L of F groups. This increase has already been demonstrated in studies with approximate F doses in organs such as the liver and kidney [11] and cerebellum [35]. The measurement of TBARS levels is one of the most relevant methods for assessing oxidative damage in lipids [29, 36]. Through it, it is possible to quantify substances generated as byproducts of tissue lipid peroxidation [29] that act as indicators of the occurrence of oxidative stress. It is known that F is able to inhibit the activity of antioxidant enzymes, such as SOD, to change the level of GSH, and to cause excessive production of ROS in the mitochondrion, leading to lipid peroxidation and cell apoptosis. Therefore, the elevation of TBARS levels in the groups exposed to F indicates the induction of lipid peroxidation and, therewith, impairment of cell structure and biological function due to stress.

The balance of oxidative biochemistry is closely related to the cellular proteostasis network. The behavior of functional protein groups can be influenced by the condition of stress, with alteration in the processes of synthesis, repair and protein degradation [30]. Thus, when assessing the proteomic profile of glandular tissue, modifications were found in the modulation of protein functional groups related to oxidative stress, such as "Response to stress" and "Organonitrogen compound metabolic process”. Not by chance, ORA results showed an over-representation of protein functional categories involved in protection against fluorine-induced stress, the chaperones. ORA is an enrichment analysis used to detect whether gene sets, pathways or biological functions are over-represented within a list of ontological categories with differences in expression [37].

According to ORA analysis, there was an increase expression of *Heat shock-related 70 kDa protein 2* (P17156) and *Heat shock 70 kDa protein 1A* (Q61696), in the 10 mg/L of F group. Heat shock proteins are chaperones involved in a wide variety of cellular processes, including protecting the proteome from stress, folding and transporting newly synthesized polypeptides, and activating proteolysis of poorly folded proteins (Uniprot). Moreover, there was upregulation of *Endoplasmic reticulum chaperone BiP* (P20029) in both exposed groups. The binding proteins (BiP) are chaperones that are involved in the correct folding of proteins and degradation of misfolded proteins (Uniprot). Our findings are reinforced by studies that report up regulation of BiP [38] and increased expression of the heat shock protein Hsp70 [39], as a result of exposure to F.

Another group of proteins whose expression has been modified was mitogen-activated protein kinase *(*MAPK). The *MAPK* 1 (P63085) and *MPK 3* (Q63844) were down-regulated in both F concentrations, and *MAPK 8* (Q91Y86) was downregulated only in the 50 mg/L of F group. MAPK act on phosphorylation a broad range of proteins involved in multiple biological processes (Uniprot). Studies report that F induces oxidative stress through the activation of MAPK signaling pathways by the phosphorylation of Jun N-terminal kinase (JNK) and extracellular signal-regulated protein kinase (ERK) [40, 41]. It is known that JNK and ERK are two family members of MAPK essential for the regulation of cell growth and apoptosis [41], therefore when interacting with this pathway, F is able to interfere in these cellular processes.

The cyclic phosphorylation/dephosphorylation process of MAPs is especially important in the conversion of interphase cells and stable microtubules to mitotic cells and dynamic microtubules [42]. Considering that F may act as a phosphatase inhibitor, it is suggested that after treatment with F, the stability of the microtubules is subject to alterations. In the hippocampus of mice that received 100 mgF/L for 60 days, a significant decrease in the expression of Tubα1a and Tubβ2a was observed, in addition to microtubule fragmentation [43]. In this study, protein categories related to the microtubular system dynamics changed in expression, representing 21% of the proteins enriched in ORA analysis. A decreased tubulin expression and an upregulation of actin was observed in both the 10 and 50mg/L of F groups.

Oxidative changes can also result, in addition to modulation of the protein expression pattern, in damage to nucleic acids, inducing DNA strand fragmentation or modification and loss of bases [44]. Evidence suggests that the genetic damage induced by F can be unleashed by mechanisms of oxidative stress and mitochondrial damage [45]. However, there is no consensus about the possible effects of F exposure on genetic material. There are studies suggesting inhibition or alteration of the mitotic pattern and development of chromosomal aberrations in rats receiving relatively low doses of F, ranging from 12 to 15 mgF/L in drinking water over a period of 1 to 3 months [44, 46]. Conversely other authors have shown no evidence of genotoxicity after exposure to F in rat blood cells [47].

In view of these controversial results, the present study sought to investigate possible damage to the nuclear component based on the knowledge that there was a biochemical imbalance and proteomic changes triggered by exposure to F. Nevertheless, from the evaluated parameters, no damage was observed to the integrity of the genetic material in the glandular tissue. It is suggested that the exposure model was not able to induce damage due to the adaptive response of the antioxidant defense system.

In summary, the findings of this study suggest that F ingestion induced oxidative stress as evidenced by the increase of lipid peroxidation. Furthermore, increased GSH content in the groups receiving F indicates an attempt to respond to stimuli with an increase in antioxidant response. The maintenance of the levels of TEAC, in turn, suggests the presence of compensatory mechanisms acting to reestablish cellular redox balance. The expression changes of chaperones and MPKA indicate a stress-induced modification of the glandular proteome due to F exposure with repercussions on the cell cytoskeleton. However, the genetic content has not suffered impairment. It is worth highlighting that the results do not support that these alterations produced modifications in the salivary composition. Thus, studies that evaluate the product of these glands are necessary to better understand the effects of F on the glands and saliva.

## 5. Conclusion

It was verified that long-term exposure to 10 and 50 mg/L concentrations of F was able to change the biochemical parameters and protein modulation of the parotid glands without interfering with the integrity of DNA and tissue structure. These findings reiterate the importance of the external control and rationalization of F use to ensure the preventive effects, reducing the environmental risk.

## Supporting information

S1 Table Global proteomic profile of mice parotid glands exposed to 10 mgF/L in comparison to the control group. (DOCX)

S2 Table Global proteomic profile of mice parotid glands exposed to 50 mgF/L in comparison to the control group. (DOCX)

S3 Table Proteins identified according over-representation analysis (ORA) in mice parotid gland, after 60 days of fluoride exposure. (DOCX)

S1 Graphical abstract (TIF)
