# Supplementary material for: Effects of long-term fluoride exposure are associated with oxidative biochemistry impairment and global proteomic modulation, but not genotoxicity, in parotid glands of mice
Source: PLoS One. 2022 Jan 27;17(1):e0261252. doi: 10.1371/journal.pone.0261252 (PMC8794182; doi:10.1371/journal.pone.0261252)
Supplement: S1 Table — (DOCX) [file pone.0261252.s001.docx]

| **Supplementary Table 1**. Global proteomic profile of mice parotid glands exposed to 10 mgF/L in comparison to the control group. List of proteins with differential regulation and exclusive expression in both groups. | | | | | | |
| --- | --- | --- | --- | --- | --- | --- |
|  |  |  | **Fold Change** | |  |  |
|  | ***^a^*Access Number** | **Protein name description** | **PLGS Score** | **10mgF/L** |  |  |
|  | O08810 | 116 kDa U5 small nuclear ribonucleoprotein component | 393,74 | -0,053 |  |  |
|  | P61982 | 14-3-3 protein gamma | 332,78 | -0,053 |  |  |
|  | O70456 | 14-3-3 protein sigma | 261,21 | -0,053 |  |  |
|  | P68254 | 14-3-3 protein theta | 261,21 | -0,053 |  |  |
|  | Q8K4S1 | 1-phosphatidylinositol 4,5-bisphosphate phosphodiesterase epsilon-1 | 331,54 | + |  |  |
|  | P14685 | 26S proteasome non-ATPase regulatory subunit 3 | 66,74 | - |  |  |
|  | P62192 | 26S proteasome regulatory subunit 4 | 153,12 | - |  |  |
|  | P62196 | 26S proteasome regulatory subunit 8 | 75,64 | - |  |  |
|  | Q61733 | 28S ribosomal protein S31, mitochondrial | 118,76 | - |  |  |
|  | Q9CQ40 | 39S ribosomal protein L49, mitochondrial | 251,83 | - |  |  |
|  | P14131 | 40S ribosomal protein S16 | 421,37 | -0,053 |  |  |
|  | P63276 | 40S ribosomal protein S17 | 296,68 | 1,051 |  |  |
|  | P62270 | 40S ribosomal protein S18 | 219,48 | - |  |  |
|  | Q9CZX8 | 40S ribosomal protein S19 | 807,45 | + |  |  |
|  | P62858 | 40S ribosomal protein S28 | 968,59 | 1,051 |  |  |
|  | P62908 | 40S ribosomal protein S3 | 1267,99 | 1,051 |  |  |
|  | P97351 | 40S ribosomal protein S3a | 350,31 | -0,053 |  |  |
|  | P62702 | 40S ribosomal protein S4, X isoform | 99,01 | 1,051 |  |  |
|  | P62754 | 40S ribosomal protein S6 | 117,39 | + |  |  |
|  | P62082 | 40S ribosomal protein S7 | 1176,69 | -0,053 |  |  |
|  | P62242 | 40S ribosomal protein S8 | 122,1 | -0,053 |  |  |
|  | P70290 | 55 kDa erythrocyte membrane protein | 308,48 | - |  |  |
|  | P47955 | 60S acidic ribosomal protein P1 | 7820,38 | 1,051 |  |  |
|  | P99027 | 60S acidic ribosomal protein P2 | 1189,82 | -0,053 |  |  |
|  | Q6ZWV3 | 60S ribosomal protein L10 | 169,22 | - |  |  |
|  | P86048 | 60S ribosomal protein L10-like | 169,22 | - |  |  |
|  | P35979 | 60S ribosomal protein L12 | 995,41 | -0,053 |  |  |
|  | P67984 | 60S ribosomal protein L22 | 751,95 | -0,053 |  |  |
|  | P62830 | 60S ribosomal protein L23 | 1000,17 | - |  |  |
|  | P14115 | 60S ribosomal protein L27a | 363,65 | + |  |  |
|  | P62889 | 60S ribosomal protein L30 | 188,13 | 1,051 |  |  |
|  | P47911 | 60S ribosomal protein L6 | 94,42 | + |  |  |
|  | P62918 | 60S ribosomal protein L8 | 516,46 | + |  |  |
|  | P20029 | 78 kDa glucose-regulated protein | 118,66 | 1,051 |  |  |
|  | Q8CBW3 | Abl interactor 1 | 68,37 | - |  |  |
|  | Q99NB1 | Acetyl-coenzyme A synthetase 2-like, mitochondrial | 107,79 | + |  |  |
|  | Q91XA9 | Acidic mammalian chitinase | 4212,14 | -0,053 |  |  |
|  | Q99KI0 | Aconitate hydratase, mitochondrial | 219,08 | 1,051 |  |  |
|  | Q80YS6 | Actin filament-associated protein 1 | 106,6 | - |  |  |
|  | P68134 | Actin, alpha skeletal muscle | 809,78 | 1,051 |  |  |
|  | P62737 | Actin, aortic smooth muscle | 809,78 | 1,051 |  |  |
|  | Q8K4G5 | Actin-binding LIM protein 1 | 256,25 | + |  |  |
|  | Q5SSL4 | Active breakpoint cluster region-related protein | 99,21 | + |  |  |
|  | Q8JZN5 | Acyl-CoA dehydrogenase family member 9, mitochondrial | 153,3 | - |  |  |
|  | Q8VCW8 | Acyl-CoA synthetase family member 2, mitochondrial | 196,02 | + |  |  |
|  | Q920P5 | Adenylate kinase isoenzyme 5 | 177,48 | + |  |  |
|  | Q64277 | ADP-ribosyl cyclase/cyclic ADP-ribose hydrolase 2 | 157,43 | + |  |  |
|  | P61750 | ADP-ribosylation factor 4 | 571,29 | -0,053 |  |  |
|  | P84084 | ADP-ribosylation factor 5 | 438,78 | -0,053 |  |  |
|  | P00329 | Alcohol dehydrogenase 1 | 233,11 | -0,053 |  |  |
|  | P47739 | Aldehyde dehydrogenase, dimeric NADP-preferring | 117,27 | - |  |  |
|  | Q5SGK3 | Aldehyde oxidase 2 | 118,13 | - |  |  |
|  | P45376 | Aldose reductase | 194,76 | -0,053 |  |  |
|  | Q812G0 | Alpha-1,3-mannosyl-glycoprotein 4-beta-N-acetylglucosaminyltransferase A | 142,71 | - |  |  |
|  | Q14BT6 | Alpha-1,4-N-acetylglucosaminyltransferase | 251,05 | + |  |  |
|  | P07758 | Alpha-1-antitrypsin 1-1 | 82,14 | + |  |  |
|  | P22599 | Alpha-1-antitrypsin 1-2 | 172,98 | + |  |  |
|  | Q00896 | Alpha-1-antitrypsin 1-3 | 82,14 | + |  |  |
|  | Q00897 | Alpha-1-antitrypsin 1-4 | 82,14 | + |  |  |
|  | Q7TPR4 | Alpha-actinin-1 | 72,08 | + |  |  |
|  | Q9JI91 | Alpha-actinin-2 | 69,23 | + |  |  |
|  | Q9QYC0 | Alpha-adducin | 259,69 | - |  |  |
|  | Q9DBF1 | Alpha-aminoadipic semialdehyde dehydrogenase | 227,3 | -0,053 |  |  |
|  | P00687 | Alpha-amylase 1 | 290,35 | -0,053 |  |  |
|  | P27046 | Alpha-mannosidase 2 | 117,89 | - |  |  |
|  | Q8VCT3 | Aminopeptidase B | 128,57 | + |  |  |
|  | Q8WTY4 | Anamorsin | 193,06 | - |  |  |
|  | D0G895 | Angiotensin-converting enzyme-like protein Ace3 | 83,79 | + |  |  |
|  | Q02357 | Ankyrin-1 | 88,37 | + |  |  |
|  | P48036 | Annexin A5 | 326,24 | -0,053 |  |  |
|  | P14824 | Annexin A6 | 153,49 | -0,053 |  |  |
|  | O88312 | Anterior gradient protein 2 homolog | 222,29 | 1,051 |  |  |
|  | P08226 | Apolipoprotein E | 342,94 | - |  |  |
|  | Q8CG79 | Apoptosis-stimulating of p53 protein 2 | 77,34 | - |  |  |
|  | Q61176 | Arginase-1 | 181,2 | + |  |  |
|  | P24721 | Asialoglycoprotein receptor 2 | 159,22 | + |  |  |
|  | Q922B2 | Aspartate--tRNA ligase, cytoplasmic | 78,94 | + |  |  |
|  | Q9Z2W0 | Aspartyl aminopeptidase | 207,39 | + |  |  |
|  | Q9CQQ7 | ATP synthase F(0) complex subunit B1, mitochondrial | 291,45 | + |  |  |
|  | P97450 | ATP synthase-coupling factor 6, mitochondrial | 145,19 | + |  |  |
|  | Q91V92 | ATP-citrate synthase | 92,4 | - |  |  |
|  | Q8VDW0 | ATP-dependent RNA helicase DDX39A | 289,77 | + |  |  |
|  | Q62167 | ATP-dependent RNA helicase DDX3X | 99,7 | + |  |  |
|  | Q14BI7 | ATP-dependent RNA helicase TDRD9 | 84,27 | + |  |  |
|  | P86174 | BEN domain-containing protein 4 | 104,8 | - |  |  |
|  | Q8BFZ3 | Beta-actin-like protein 2 | 1101,7 | 1,051 |  |  |
|  | Q99MK8 | Beta-adrenergic receptor kinase 1 | 201,92 | - |  |  |
|  | P21550 | Beta-enolase | 208,66 | 1,051 |  |  |
|  | P20060 | Beta-hexosaminidase subunit beta | 248,42 | + |  |  |
|  | Q99N32 | Beta-klotho | 112,67 | - |  |  |
|  | P01139 | Beta-nerve growth factor | 608,79 | + |  |  |
|  | Q91Z96 | BMP-2-inducible protein kinase | 94,4 | + |  |  |
|  | P24288 | Branched-chain-amino-acid aminotransferase, cytosolic | 81,93 | - |  |  |
|  | Q8BMB0 | BRCA2-interacting transcriptional repressor EMSY | 92,12 | - |  |  |
|  | Q921C3 | Bromodomain and WD repeat-containing protein 1 | 193,62 | - |  |  |
|  | Q52KB6 | C2 domain-containing protein 3 | 54,22 | + |  |  |
|  | P70408 | Cadherin-10 | 144,84 | - |  |  |
|  | Q5RJH3 | Cadherin-12 | 96,73 | + |  |  |
|  | P97291 | Cadherin-8 | 175,77 | - |  |  |
|  | Q8BG22 | Calcium-activated chloride channel regulator 2 | 128,37 | - |  |  |
|  | Q6Q473 | Calcium-activated chloride channel regulator 4A | 80,3 | + |  |  |
|  | Q9QXX4 | Calcium-binding mitochondrial carrier protein Aralar2 | 149,86 | - |  |  |
|  | P14211 | Calreticulin | 177,37 | -0,053 |  |  |
|  | O09165 | Calsequestrin-1 | 264,44 | + |  |  |
|  | Q01063 | cAMP-specific 3',5'-cyclic phosphodiesterase 4D | 96,36 | + |  |  |
|  | Q9Z0H8 | CAP-Gly domain-containing linker protein 2 | 109,8 | - |  |  |
|  | P00920 | Carbonic anhydrase 2 | 141,3 | - |  |  |
|  | P16015 | Carbonic anhydrase 3 | 643,78 | -0,053 |  |  |
|  | P61215 | Carbonic anhydrase-related protein 10 | 195,56 | + |  |  |
|  | P48758 | Carbonyl reductase [NADPH] 1 | 371,22 | -0,053 |  |  |
|  | A2AIV8 | Caspase recruitment domain-containing protein 9 | 141,16 | - |  |  |
|  | P26231 | Catenin alpha-1 | 85,58 | + |  |  |
|  | Q61301 | Catenin alpha-2 | 113,92 | + |  |  |
|  | Q02248 | Catenin beta-1 | 230,56 | - |  |  |
|  | Q8K1J6 | CCA tRNA nucleotidyltransferase 1, mitochondrial | 129,97 | - |  |  |
|  | Q8CII2 | Cell division cycle protein 123 homolog | 518,28 | - |  |  |
|  | Q8CB62 | Centrobin | 166,2 | - |  |  |
|  | Q9CZW2 | Centromere protein N | 219,07 | - |  |  |
|  | Q9CZX2 | Centrosomal protein of 89 kDa | 131,8 | - |  |  |
|  | E9Q309 | Centrosome-associated protein 350 | 115,49 | + |  |  |
|  | A2A6T1 | Cerebellar degeneration-related protein 2-like | 215,81 | - |  |  |
|  | Q8R090 | Chromaffin granule amine transporter | 82,74 | - |  |  |
|  | Q68FD5 | Clathrin heavy chain 1 | 280,5 | 1,051 |  |  |
|  | Q8VBZ3 | Cleft lip and palate transmembrane protein 1 homolog | 88,33 | + |  |  |
|  | Q8CIE6 | Coatomer subunit alpha | 112,01 | -0,053 |  |  |
|  | Q9QZE5 | Coatomer subunit gamma-1 | 172,41 | -0,053 |  |  |
|  | Q3URS9 | Coiled-coil domain-containing protein 51 | 136,36 | - |  |  |
|  | Q8CDM4 | Coiled-coil domain-containing protein 73 | 179,91 | - |  |  |
|  | P08121 | Collagen alpha-1(III) chain | 150,39 | + |  |  |
|  | Q61245 | Collagen alpha-1(XI) chain | 98,79 | + |  |  |
|  | Q9QZS0 | Collagen alpha-3(IV) chain | 817,58 | + |  |  |
|  | P01027 | Complement C3 | 112,45 | - |  |  |
|  | P06683 | Complement component C9 | 407,82 | + |  |  |
|  | Q8R3I3 | Conserved oligomeric Golgi complex subunit 6 | 335,28 | + |  |  |
|  | Q0V8T8 | Contactin-associated protein like 5-2 | 135,06 | + |  |  |
|  | P59108 | Copine-2 | 72,91 | + |  |  |
|  | P07310 | Creatine kinase M-type | 172,46 | 1,051 |  |  |
|  | P30275 | Creatine kinase U-type, mitochondrial | 118,63 | - |  |  |
|  | Q6ZQ38 | Cullin-associated NEDD8-dissociated protein 1 | 75,46 | - |  |  |
|  | P49919 | Cyclin-dependent kinase inhibitor 1C | 158,58 | + |  |  |
|  | Q9JM84 | Cystatin 10 | 930,37 | 1,051 |  |  |
|  | Q03401 | Cysteine-rich secretory protein 1 | 100,04 | 1,051 |  |  |
|  | P19783 | Cytochrome c oxidase subunit 4 isoform 1, mitochondrial | 270,65 | - |  |  |
|  | P12787 | Cytochrome c oxidase subunit 5A, mitochondrial | 129,71 | 1,051 |  |  |
|  | P56391 | Cytochrome c oxidase subunit 6B1 | 332,38 | + |  |  |
|  | P56657 | Cytochrome P450 2C40 | 111,58 | + |  |  |
|  | Q91WL5 | Cytochrome P450 4A12A | 121,54 | - |  |  |
|  | P28271 | Cytoplasmic aconitate hydratase | 109,28 | - |  |  |
|  | Q7TN99 | Cytoplasmic polyadenylation element-binding protein 3 | 110,31 | + |  |  |
|  | Q3V1H1 | Cytoskeleton-associated protein 2 | 104,35 | + |  |  |
|  | Q8K3G9 | DCC-interacting protein 13-beta | 68,43 | - |  |  |
|  | O88843 | Death domain-containing protein CRADD | 284,09 | - |  |  |
|  | P28654 | Decorin | 255,04 | 1,051 |  |  |
|  | Q99KU1 | Dehydrodolichyl diphosphate synthase complex subunit Dhdds | 63,58 | - |  |  |
|  | Q9Z110 | Delta-1-pyrroline-5-carboxylate synthase | 178,2 | + |  |  |
|  | Q9WV69 | Dematin | 105,25 | + |  |  |
|  | Q8C4S8 | DENN domain-containing protein 2A | 100,97 | + |  |  |
|  | A2RSQ0 | DENN domain-containing protein 5B | 305,34 | + |  |  |
|  | P31001 | Desmin | 74,61 | - |  |  |
|  | Q9R0P5 | Destrin | 357,92 | + |  |  |
|  | Q8BTT6 | Digestive organ expansion factor homolog | 157,03 | + |  |  |
|  | Q8BWT5 | Disco-interacting protein 2 homolog A | 164,95 | + |  |  |
|  | Q8K4R9 | Disks large-associated protein 5 | 125,32 | - |  |  |
|  | Q6P6J4 | DNA oxidative demethylase ALKBH2 | 970,49 | - |  |  |
|  | Q7TQ07 | DNA polymerase nu | 79,2 | - |  |  |
|  | Q6PFE3 | DNA repair and recombination protein RAD54B | 216,83 | - |  |  |
|  | Q6ZQF0 | DNA topoisomerase 2-binding protein 1 | 164,27 | 1,051 |  |  |
|  | P52432 | DNA-directed RNA polymerases I and III subunit RPAC1 | 445,95 | - |  |  |
|  | Q99KV1 | DnaJ homolog subfamily B member 11 | 131,4 | -0,053 |  |  |
|  | Q9R022 | DnaJ homolog subfamily C member 12 | 257,48 | - |  |  |
|  | Q91YW3 | DnaJ homolog subfamily C member 3 | 372,09 | -0,053 |  |  |
|  | O54734 | Dolichyl-diphosphooligosaccharide--protein glycosyltransferase 48 kDa subunit | 665,29 | -0,053 |  |  |
|  | Q4VA61 | Down syndrome cell adhesion molecule-like protein 1 homolog | 142,69 | - |  |  |
|  | Q6XUX1 | Dual serine/threonine and tyrosine protein kinase | 288,08 | - |  |  |
|  | Q3V0Q1 | Dynein heavy chain 12, axonemal | 108,45 | - |  |  |
|  | Q9CZ00 | Dysbindin domain-containing protein 1 | 211,32 | + |  |  |
|  | Q8CHI8 | E1A-binding protein p400 | 110,66 | - |  |  |
|  | Q8C669 | E3 ubiquitin-protein ligase pellino homolog 1 | 102,01 | + |  |  |
|  | O35445 | E3 ubiquitin-protein ligase RNF5 | 193,81 | - |  |  |
|  | Q6PCX9 | E3 ubiquitin-protein ligase TRIM37 | 187,4 | + |  |  |
|  | Q38HM4 | E3 ubiquitin-protein ligase TRIM63 | 85,14 | - |  |  |
|  | Q8BL66 | Early endosome antigen 1 | 81,99 | - |  |  |
|  | P06802 | Ectonucleotide pyrophosphatase/phosphodiesterase family member 1 | 146,2 | + |  |  |
|  | Q99LC5 | Electron transfer flavoprotein subunit alpha, mitochondrial | 165,06 | 1,051 |  |  |
|  | Q99MI1 | ELKS/Rab6-interacting/CAST family member 1 | 80,72 | 1,051 |  |  |
|  | P57680 | Ellis-van Creveld syndrome protein homolog | 170,42 | - |  |  |
|  | P10126 | Elongation factor 1-alpha 1 | 441,3 | 1,051 |  |  |
|  | P62631 | Elongation factor 1-alpha 2 | 2869,59 | 1,051 |  |  |
|  | Q9D8N0 | Elongation factor 1-gamma | 531,83 | -0,053 |  |  |
|  | P58252 | Elongation factor 2 | 698,84 | -0,053 |  |  |
|  | Q8K482 | EMILIN-2 | 85,39 | + |  |  |
|  | Q9D1Q6 | Endoplasmic reticulum resident protein 44 | 834,97 | 1,051 |  |  |
|  | Q9DC16 | Endoplasmic reticulum-Golgi intermediate compartment protein 1 | 314,28 | - |  |  |
|  | O35393 | Ephrin-B3 | 186,07 | + |  |  |
|  | P36368 | Epidermal growth factor-binding protein type B | 7223,62 | + |  |  |
|  | Q3US41 | Epithelial splicing regulatory protein 1 | 127,47 | - |  |  |
|  | Q6IE26 | Epoxide hydrolase 4 | 175,66 | -0,053 |  |  |
|  | Q9JIM1 | Equilibrative nucleoside transporter 1 | 58,97 | + |  |  |
|  | Q9EQ06 | Estradiol 17-beta-dehydrogenase 11 | 154,52 | + |  |  |
|  | P60843 | Eukaryotic initiation factor 4A-I | 492,27 | -0,053 |  |  |
|  | Q91VC3 | Eukaryotic initiation factor 4A-III | 178,27 | -0,053 |  |  |
|  | P63242 | Eukaryotic translation initiation factor 5A-1 | 245,96 | + |  |  |
|  | Q8BGY2 | Eukaryotic translation initiation factor 5A-2 | 245,96 | + |  |  |
|  | Q9CXP9 | Exonuclease V | 293,15 | - |  |  |
|  | Q9JHI7 | Exosome complex component RRP45 | 384,07 | - |  |  |
|  | Q9CRT8 | Exportin-T | 129,63 | + |  |  |
|  | Q9D4K4 | FANCD2 opposite strand protein | 144,01 | - |  |  |
|  | Q80V62 | Fanconi anemia group D2 protein homolog | 196,68 | + |  |  |
|  | Q920E5 | Farnesyl pyrophosphate synthase | 90,34 | - |  |  |
|  | Q05816 | Fatty acid-binding protein, epidermal | 521,36 | + |  |  |
|  | Q922J9 | Fatty acyl-CoA reductase 1 | 90,63 | + |  |  |
|  | Q3USJ8 | F-BAR and double SH3 domains protein 2 | 93,82 | - |  |  |
|  | Q6DIA9 | F-box only protein 27 | 165,91 | + |  |  |
|  | Q9QZN1 | F-box/LRR-repeat protein 17 | 264,37 | + |  |  |
|  | Q3UY23 | Ferredoxin-fold anticodon-binding domain-containing protein 1 homolog | 132,33 | - |  |  |
|  | P29391 | Ferritin light chain 1 | 689,46 | -0,053 |  |  |
|  | Q8VCM7 | Fibrinogen gamma chain | 107,03 | + |  |  |
|  | O08696 | Forkhead box protein M1 | 268,72 | + |  |  |
|  | P05063 | Fructose-bisphosphate aldolase C | 856,43 | + |  |  |
|  | P97807 | Fumarate hydratase, mitochondrial | 170,1 | + |  |  |
|  | Q5RJY2 | G2/M phase-specific E3 ubiquitin-protein ligase | 57,13 | - |  |  |
|  | Q8QZW7 | Gamma-aminobutyric acid receptor subunit pi | 273,51 | + |  |  |
|  | Q3TCV3 | Gamma-secretase-activating protein | 182,23 | + |  |  |
|  | Q01231 | Gap junction alpha-5 protein | 149,2 | + |  |  |
|  | Q5SSG4 | GAS2-like protein 2 | 239,61 | - |  |  |
|  | Q5Y4Y6 | Gasdermin-A3 | 209,65 | + |  |  |
|  | P23591 | GDP-L-fucose synthase | 466 | + |  |  |
|  | Q9JI57 | General transcription factor II-I repeat domain-containing protein 1 | 161,76 | + |  |  |
|  | Q9Z1Z0 | General vesicular transport factor p115 | 183,09 | -0,053 |  |  |
|  | Q9JHK4 | Geranylgeranyl transferase type-2 subunit alpha | 98,14 | + |  |  |
|  | Q9CQI3 | Glia maturation factor beta | 122,84 | + |  |  |
|  | Q01098 | Glutamate receptor ionotropic, NMDA 2C | 76,81 | + |  |  |
|  | P10649 | Glutathione S-transferase Mu 1 | 162,11 | -0,053 |  |  |
|  | Q64467 | Glyceraldehyde-3-phosphate dehydrogenase, testis-specific | 111,45 | -0,053 |  |  |
|  | Q61586 | Glycerol-3-phosphate acyltransferase 1, mitochondrial | 83,07 | + |  |  |
|  | Q9QXF8 | Glycine N-methyltransferase | 134,87 | + |  |  |
|  | Q9R062 | Glycogenin-1 | 138,89 | + |  |  |
|  | Q91Z53 | Glyoxylate reductase/hydroxypyruvate reductase | 269,57 | + |  |  |
|  | O35166 | Golgi SNAP receptor complex member 2 | 284,76 | -0,053 |  |  |
|  | Q9JJI6 | GPI ethanolamine phosphate transferase 3 | 127,61 | - |  |  |
|  | Q8C0K5 | Graves disease carrier protein homolog | 104,36 | - |  |  |
|  | Q60780 | Growth arrest-specific protein 7 | 220,11 | + |  |  |
|  | Q8VDV3 | Guanine nucleotide exchange factor for Rab-3A | 214,11 | + |  |  |
|  | P18872 | Guanine nucleotide-binding protein G(o) subunit alpha | 134,18 | + |  |  |
|  | Q9ESX5 | H/ACA ribonucleoprotein complex subunit 4 | 147,55 | - |  |  |
|  | Q61696 | Heat shock 70 kDa protein 1A | 783 | 1,051 |  |  |
|  | P17879 | Heat shock 70 kDa protein 1B | 787,2 | 1,051 |  |  |
|  | P16627 | Heat shock 70 kDa protein 1-like | 850,23 | -0,053 |  |  |
|  | P17156 | Heat shock-related 70 kDa protein 2 | 965,94 | 1,051 |  |  |
|  | P01942 | Hemoglobin subunit alpha | 900,76 | -0,053 |  |  |
|  | P02088 | Hemoglobin subunit beta-1 | 2039,06 | 1,051 |  |  |
|  | P02089 | Hemoglobin subunit beta-2 | 1031,21 | -0,053 |  |  |
|  | P06467 | Hemoglobin subunit zeta | 291,89 | 1,051 |  |  |
|  | Q91X72 | Hemopexin | 181,63 | -0,053 |  |  |
|  | Q8BG05 | Heterogeneous nuclear ribonucleoprotein A3 | 125,91 | + |  |  |
|  | P61979 | Heterogeneous nuclear ribonucleoprotein K | 382,59 | + |  |  |
|  | Q8R081 | Heterogeneous nuclear ribonucleoprotein L | 144,75 | + |  |  |
|  | Q8VEK3 | Heterogeneous nuclear ribonucleoprotein U | 195,44 | - |  |  |
|  | O88569 | Heterogeneous nuclear ribonucleoproteins A2/B1 | 324,33 | + |  |  |
|  | E9Q4S1 | High affinity cAMP-specific and IBMX-insensitive 3',5'-cyclic phosphodiesterase 8B | 53,32 | + |  |  |
|  | P70349 | Histidine triad nucleotide-binding protein 1 | 349,21 | + |  |  |
|  | B2RWS6 | Histone acetyltransferase p300 | 183,31 | + |  |  |
|  | Q9Z2V5 | Histone deacetylase 6 | 196,4 | + |  |  |
|  | Q8C2B3 | Histone deacetylase 7 | 92,48 | + |  |  |
|  | Q8CGP5 | Histone H2A type 1-F | 20828,94 | -0,053 |  |  |
|  | Q8CGP6 | Histone H2A type 1-H | 20828,94 | -0,053 |  |  |
|  | Q8CGP7 | Histone H2A type 1-K | 20828,94 | -0,053 |  |  |
|  | Q6GSS7 | Histone H2A type 2-A | 20828,94 | -0,053 |  |  |
|  | Q64522 | Histone H2A type 2-B | 2548,16 | -0,053 |  |  |
|  | Q64523 | Histone H2A type 2-C | 20828,94 | -0,053 |  |  |
|  | Q8BFU2 | Histone H2A type 3 | 20828,94 | -0,053 |  |  |
|  | Q8R1M2 | Histone H2A.J | 20828,94 | -0,053 |  |  |
|  | Q3THW5 | Histone H2A.V | 2696,72 | -0,053 |  |  |
|  | P0C0S6 | Histone H2A.Z | 2696,72 | -0,053 |  |  |
|  | P27661 | Histone H2AX | 2696,72 | -0,053 |  |  |
|  | Q64475 | Histone H2B type 1-B | 10972,62 | -0,053 |  |  |
|  | Q6ZWY9 | Histone H2B type 1-C/E/G | 10972,62 | -0,053 |  |  |
|  | P10853 | Histone H2B type 1-F/J/L | 10972,62 | -0,053 |  |  |
|  | Q64478 | Histone H2B type 1-H | 10972,62 | -0,053 |  |  |
|  | Q8CGP1 | Histone H2B type 1-K | 10972,62 | -0,053 |  |  |
|  | P10854 | Histone H2B type 1-M | 10972,62 | -0,053 |  |  |
|  | Q8CGP2 | Histone H2B type 1-P | 10972,62 | -0,053 |  |  |
|  | Q64525 | Histone H2B type 2-B | 10972,62 | -0,053 |  |  |
|  | Q64524 | Histone H2B type 2-E | 9182,73 | -0,053 |  |  |
|  | Q9D2U9 | Histone H2B type 3-A | 9182,73 | -0,053 |  |  |
|  | Q8CGP0 | Histone H2B type 3-B | 9182,73 | -0,053 |  |  |
|  | P84228 | Histone H3.2 | 1843,33 | 1,051 |  |  |
|  | P70351 | Histone-lysine N-methyltransferase EZH1 | 134,33 | + |  |  |
|  | Q61188 | Histone-lysine N-methyltransferase EZH2 | 127,8 | + |  |  |
|  | E9Q5F9 | Histone-lysine N-methyltransferase SETD2 | 91,74 | - |  |  |
|  | Q8VD75 | Huntingtin-interacting protein 1 | 87,29 | + |  |  |
|  | P01878 | Ig alpha chain C region | 822,98 | -0,053 |  |  |
|  | P01868 | Ig gamma-1 chain C region secreted form | 216,22 | + |  |  |
|  | P01869 | Ig gamma-1 chain C region, membrane-bound form | 216,22 | + |  |  |
|  | P01864 | Ig gamma-2A chain C region secreted form | 178,01 | -0,053 |  |  |
|  | P01863 | Ig gamma-2A chain C region, A allele | 157,39 | -0,053 |  |  |
|  | P01865 | Ig gamma-2A chain C region, membrane-bound form | 157,39 | -0,053 |  |  |
|  | P01867 | Ig gamma-2B chain C region | 518,82 | -0,053 |  |  |
|  | P01837 | Ig kappa chain C region | 668,32 | - |  |  |
|  | P01723 | Ig lambda-1 chain V region | 372,51 | + |  |  |
|  | P01727 | Ig lambda-1 chain V region S43 | 372,51 | + |  |  |
|  | Q5RKR3 | Immunoglobulin superfamily containing leucine-rich repeat protein 2 | 463,03 | - |  |  |
|  | Q3V1M1 | Immunoglobulin superfamily member 10 | 257,97 | + |  |  |
|  | Q8K0C1 | Importin-13 | 86,6 | 1,051 |  |  |
|  | Q7M6U3 | Inactive serine/threonine-protein kinase TEX14 | 116,91 | - |  |  |
|  | P15975 | Inactive ubiquitin carboxyl-terminal hydrolase 53 | 182,27 | - |  |  |
|  | Q9Z329 | Inositol 1,4,5-trisphosphate receptor type 2 | 94,88 | + |  |  |
|  | B8JK39 | Integrin alpha-9 | 112,59 | + |  |  |
|  | Q9QZ85 | Interferon-inducible GTPase 1 | 117,33 | + |  |  |
|  | Q9Z1X4 | Interleukin enhancer-binding factor 3 | 112,37 | - |  |  |
|  | Q9Z0R4 | Intersectin-1 | 292,55 | 1,051 |  |  |
|  | Q9CUL5 | IQ and AAA domain-containing protein 1 | 101,07 | + |  |  |
|  | P85094 | Isochorismatase domain-containing protein 2A | 1368,09 | -0,053 |  |  |
|  | O88844 | Isocitrate dehydrogenase [NADP] cytoplasmic | 446,83 | - |  |  |
|  | P00755 | Kallikrein 1-related peptidase b1 | 463,54 | + |  |  |
|  | P15946 | Kallikrein 1-related peptidase b11 | 503,96 | + |  |  |
|  | P04071 | Kallikrein 1-related peptidase b16 | 9641,6 | + |  |  |
|  | Q61759 | Kallikrein 1-related peptidase b21 | 8995,33 | + |  |  |
|  | P15948 | Kallikrein 1-related peptidase b22 | 206,71 | 1,051 |  |  |
|  | Q61754 | Kallikrein 1-related peptidase b24 | 6010,96 | + |  |  |
|  | Q9JM71 | Kallikrein 1-related peptidase b27 | 7698,94 | + |  |  |
|  | P00756 | Kallikrein 1-related peptidase b3 | 6016,17 | + |  |  |
|  | P15945 | Kallikrein 1-related peptidase b5 | 10494,55 | + |  |  |
|  | P07628 | Kallikrein 1-related peptidase b8 | 6916,1 | + |  |  |
|  | P15949 | Kallikrein 1-related peptidase b9 | 522,51 | + |  |  |
|  | P00757 | Kallikrein 1-related peptidase-like b4 | 1770,53 | + |  |  |
|  | P15947 | Kallikrein-1 | 11783,46 | + |  |  |
|  | Q9Z2X8 | Kelch-like ECH-associated protein 1 | 88,97 | - |  |  |
|  | B1AQ75 | Keratin, type I cuticular Ha6 | 192,32 | -0,053 |  |  |
|  | P05784 | Keratin, type I cytoskeletal 18 | 91,52 | -0,053 |  |  |
|  | Q9D312 | Keratin, type I cytoskeletal 20 | 202,72 | + |  |  |
|  | Q3TRJ4 | Keratin, type I cytoskeletal 26 | 147,98 | - |  |  |
|  | Q9ERE2 | Keratin, type II cuticular Hb1 | 152,35 | -0,053 |  |  |
|  | Q99M73 | Keratin, type II cuticular Hb4 | 90,27 | -0,053 |  |  |
|  | Q9Z2T6 | Keratin, type II cuticular Hb5 | 152,35 | -0,053 |  |  |
|  | P97861 | Keratin, type II cuticular Hb6 | 152,35 | -0,053 |  |  |
|  | P04104 | Keratin, type II cytoskeletal 1 | 128,33 | + |  |  |
|  | Q3UV17 | Keratin, type II cytoskeletal 2 oral | 90,27 | -0,053 |  |  |
|  | P07744 | Keratin, type II cytoskeletal 4 | 153,27 | -0,053 |  |  |
|  | Q922U2 | Keratin, type II cytoskeletal 5 | 139,04 | -0,053 |  |  |
|  | P50446 | Keratin, type II cytoskeletal 6A | 90,27 | -0,053 |  |  |
|  | Q9R0H5 | Keratin, type II cytoskeletal 71 | 104,57 | + |  |  |
|  | Q6IFZ9 | Keratin, type II cytoskeletal 74 | 131,3 | + |  |  |
|  | Q8BGZ7 | Keratin, type II cytoskeletal 75 | 191,62 | -0,053 |  |  |
|  | Q8VED5 | Keratin, type II cytoskeletal 79 | 152,35 | -0,053 |  |  |
|  | P11679 | Keratin, type II cytoskeletal 8 | 235,39 | -0,053 |  |  |
|  | P33175 | Kinesin heavy chain isoform 5A | 74,25 | - |  |  |
|  | Q99PT9 | Kinesin-like protein KIF19 | 92,08 | + |  |  |
|  | P33173 | Kinesin-like protein KIF1A | 292,73 | + |  |  |
|  | Q80WE4 | Kinesin-like protein KIF20B | 94,01 | + |  |  |
|  | O35231 | Kinesin-like protein KIFC3 | 81,78 | + |  |  |
|  | Q99KP3 | Lambda-crystallin homolog | 164,98 | - |  |  |
|  | P02468 | Laminin subunit gamma-1 | 102,44 | - |  |  |
|  | Q8K0C4 | Lanosterol 14-alpha demethylase | 101,07 | - |  |  |
|  | Q80Y17 | Lethal(2) giant larvae protein homolog 1 | 86,9 | - |  |  |
|  | Q61809 | Leucine-rich repeat neuronal protein 1 | 119,53 | - |  |  |
|  | Q5S006 | Leucine-rich repeat serine/threonine-protein kinase 2 | 98,31 | - |  |  |
|  | Q8BZ81 | Leucine-rich repeat transmembrane neuronal protein 3 | 307,21 | + |  |  |
|  | Q3UVD5 | Leucine-rich repeat-containing G-protein coupled receptor 6 | 86,88 | - |  |  |
|  | Q8BTN6 | Leukocyte receptor cluster member 9 | 143,05 | + |  |  |
|  | Q7TMC8 | L-fucose kinase | 102,44 | + |  |  |
|  | Q9JKS4 | LIM domain-binding protein 3 | 82,83 | + |  |  |
|  | Q148V7 | LisH domain and HEAT repeat-containing protein KIAA1468 | 425,65 | + |  |  |
|  | P06151 | L-lactate dehydrogenase A chain | 227,76 | + |  |  |
|  | P35951 | Low-density lipoprotein receptor | 128,36 | - |  |  |
|  | Q9ERE7 | LRP chaperone MESD | 460,71 | + |  |  |
|  | P51885 | Lumican | 113,48 | 1,051 |  |  |
|  | P32067 | Lupus La protein homolog | 166,62 | + |  |  |
|  | P41230 | Lysine-specific demethylase 5C | 200,24 | + |  |  |
|  | O09159 | Lysosomal alpha-mannosidase | 85,71 | + |  |  |
|  | P34884 | Macrophage migration inhibitory factor | 421,77 | 1,051 |  |  |
|  | P04939 | Major urinary protein 3 | 2769,24 | 1,051 |  |  |
|  | P11590 | Major urinary protein 4 | 353,56 | 1,051 |  |  |
|  | Q8R3F5 | Malonyl-CoA-acyl carrier protein transacylase, mitochondrial | 128,42 | - |  |  |
|  | Q0PMG2 | MAM domain-containing glycosylphosphatidylinositol anchor protein 1 | 270,01 | - |  |  |
|  | Q924M7 | Mannose-6-phosphate isomerase | 289,59 | + |  |  |
|  | Q9DB40 | Mediator of RNA polymerase II transcription subunit 27 | 94,94 | - |  |  |
|  | Q9DB91 | Mediator of RNA polymerase II transcription subunit 29 | 109,23 | + |  |  |
|  | Q8BRM6 | Meiosis-specific protein MEI4 | 141,88 | - |  |  |
|  | O70423 | Membrane primary amine oxidase | 107,38 | + |  |  |
|  | Q9WVQ1 | Membrane-associated guanylate kinase, WW and PDZ domain-containing protein 2 | 116,08 | - |  |  |
|  | Q6RHR9 | Membrane-associated guanylate kinase, WW and PDZ domain-containing protein 1 | 123,58 | + |  |  |
|  | Q9CXI5 | Mesencephalic astrocyte-derived neurotrophic factor | 356,35 | -0,053 |  |  |
|  | P70669 | Metalloendopeptidase homolog PEX | 218,27 | - |  |  |
|  | Q9EQ20 | Methylmalonate-semialdehyde dehydrogenase [acylating], mitochondrial | 289,52 | 1,051 |  |  |
|  | Q8BGT6 | MICAL-like protein 1 | 96,8 | + |  |  |
|  | Q8VCF0 | Mitochondrial antiviral-signaling protein | 228,85 | + |  |  |
|  | Q9DAT5 | Mitochondrial tRNA-specific 2-thiouridylase 1 | 108,5 | + |  |  |
|  | Q920G8 | Mitoferrin-1 | 163,18 | 1,051 |  |  |
|  | P63085 | Mitogen-activated protein kinase 1 | 178,2 | - |  |  |
|  | Q63844 | Mitogen-activated protein kinase 3 | 169,41 | - |  |  |
|  | Q9ESL4 | Mitogen-activated protein kinase kinase kinase 20 | 92,37 | + |  |  |
|  | O08901 | Mitotic checkpoint serine/threonine-protein kinase BUB1 | 86,85 | + |  |  |
|  | Q9D071 | MMS19 nucleotide excision repair protein homolog | 155,93 | - |  |  |
|  | P19467 | Mucin-13 | 65,05 | - |  |  |
|  | B2RPV6 | Multimerin-1 | 83,33 | + |  |  |
|  | Q8VBX6 | Multiple PDZ domain protein | 414,32 | - |  |  |
|  | P28665 | Murinoglobulin-1 | 85,52 | -0,053 |  |  |
|  | Q60605 | Myosin light polypeptide 6 | 347,91 | 1,051 |  |  |
|  | Q3THE2 | Myosin regulatory light chain 12B | 231,93 | + |  |  |
|  | P97457 | Myosin regulatory light chain 2, skeletal muscle isoform | 1841,96 | + |  |  |
|  | Q5SX40 | Myosin-1 | 281,4 | 1,051 |  |  |
|  | Q61879 | Myosin-10 | 356,42 | + |  |  |
|  | O08638 | Myosin-11 | 102,34 | + |  |  |
|  | P13541 | Myosin-3 | 172,23 | 1,051 |  |  |
|  | Q5SX39 | Myosin-4 | 308,31 | 1,051 |  |  |
|  | Q02566 | Myosin-6 | 236,87 | 1,051 |  |  |
|  | Q91Z83 | Myosin-7 | 234,02 | 1,051 |  |  |
|  | A2AQP0 | Myosin-7B | 155,75 | 1,051 |  |  |
|  | P13542 | Myosin-8 | 296,4 | 1,051 |  |  |
|  | Q9Z2C4 | Myotubularin-related protein 1 | 260,49 | - |  |  |
|  | Q91XS1 | Myotubularin-related protein 4 | 442,61 | + |  |  |
|  | P70441 | Na(+)/H(+) exchange regulatory cofactor NHE-RF1 | 147,15 | - |  |  |
|  | Q99MJ6 | Na(+)/H(+) exchange regulatory cofactor NHE-RF4 | 88,77 | - |  |  |
|  | Q8JZV7 | N-acetylglucosamine-6-phosphate deacetylase | 142,37 | + |  |  |
|  | Q66X19 | NACHT, LRR and PYD domains-containing protein 4E | 87,88 | + |  |  |
|  | Q66X22 | NACHT, LRR and PYD domains-containing protein 9B | 138,91 | - |  |  |
|  | Q9CPP6 | NADH dehydrogenase [ubiquinone] 1 alpha subcomplex subunit 5 | 355,65 | - |  |  |
|  | Q91YT0 | NADH dehydrogenase [ubiquinone] flavoprotein 1, mitochondrial | 436,29 | + |  |  |
|  | Q8BWZ3 | N-alpha-acetyltransferase 25, NatB auxiliary subunit | 274,32 | + |  |  |
|  | P19426 | Negative elongation factor E | 195,44 | - |  |  |
|  | Q9CS84 | Neurexin-1 | 127,97 | + |  |  |
|  | P0DI97 | Neurexin-1-beta | 105,33 | + |  |  |
|  | P21661 | Neuroendocrine convertase 2 | 116,4 | + |  |  |
|  | Q3TRM4 | Neuropathy target esterase | 205,03 | + |  |  |
|  | P97333 | Neuropilin-1 | 86,67 | + |  |  |
|  | Q8BHN3 | Neutral alpha-glucosidase AB | 85,92 | -0,053 |  |  |
|  | Q9JHE3 | Neutral ceramidase | 125,69 | + |  |  |
|  | Q09014 | Neutrophil cytosol factor 1 | 111,41 | + |  |  |
|  | Q8CAF4 | NHS-like protein 1 | 167,51 | - |  |  |
|  | P10493 | Nidogen-1 | 85,92 | + |  |  |
|  | Q6ZQ12 | Ninein-like protein | 121,31 | - |  |  |
|  | O35892 | Nuclear autoantigen Sp-100 | 115 | + |  |  |
|  | Q8BJ71 | Nuclear pore complex protein Nup93 | 76,21 | - |  |  |
|  | Q60974 | Nuclear receptor corepressor 1 | 24,07 | - |  |  |
|  | Q8C163 | Nuclease EXOG, mitochondrial | 268,09 | - |  |  |
|  | Q61937 | Nucleophosmin | 132,12 | + |  |  |
|  | P15532 | Nucleoside diphosphate kinase A | 1534,19 | 1,051 |  |  |
|  | Q01768 | Nucleoside diphosphate kinase B | 136,82 | 1,051 |  |  |
|  | Q9D3H2 | Odorant-binding protein 1a | 1146,93 | 1,051 |  |  |
|  | A2AEP0 | Odorant-binding protein 1b | 83,82 | - |  |  |
|  | Q9D478 | Outer dense fiber protein 2-like | 85,89 | - |  |  |
|  | P00688 | Pancreatic alpha-amylase | 4613,28 | -0,053 |  |  |
|  | P17742 | Peptidyl-prolyl cis-trans isomerase A | 1327,07 | -0,053 |  |  |
|  | P24369 | Peptidyl-prolyl cis-trans isomerase B | 510,45 | -0,053 |  |  |
|  | P45878 | Peptidyl-prolyl cis-trans isomerase FKBP2 | 203,41 | + |  |  |
|  | Q64378 | Peptidyl-prolyl cis-trans isomerase FKBP5 | 112,05 | + |  |  |
|  | Q9DBG5 | Perilipin-3 | 128,05 | + |  |  |
|  | Q61171 | Peroxiredoxin-2 | 747,18 | -0,053 |  |  |
|  | P99029 | Peroxiredoxin-5, mitochondrial | 663,22 | + |  |  |
|  | Q2PFD7 | PH and SEC7 domain-containing protein 3 | 73,35 | - |  |  |
|  | Q8C0C7 | Phenylalanine--tRNA ligase alpha subunit | 126,12 | - |  |  |
|  | P70296 | Phosphatidylethanolamine-binding protein 1 | 897,26 | -0,053 |  |  |
|  | Q64143 | Phosphatidylinositol 3-kinase regulatory subunit gamma | 284,47 | + |  |  |
|  | O70167 | Phosphatidylinositol 4-phosphate 3-kinase C2 domain-containing subunit gamma | 127,09 | - |  |  |
|  | Q9DBJ1 | Phosphoglycerate mutase 1 | 640,48 | -0,053 |  |  |
|  | O70250 | Phosphoglycerate mutase 2 | 364,29 | + |  |  |
|  | Q3UMZ3 | Phospholipid phosphatase 5 | 214,44 | - |  |  |
|  | Q8BWJ3 | Phosphorylase b kinase regulatory subunit alpha, liver isoform | 83,53 | + |  |  |
|  | Q9QY23 | Plakophilin-3 | 342,45 | + |  |  |
|  | Q68FH0 | Plakophilin-4 | 246,2 | + |  |  |
|  | Q61233 | Plastin-2 | 150,29 | 1,051 |  |  |
|  | Q08481 | Platelet endothelial cell adhesion molecule | 113,67 | + |  |  |
|  | Q9QXS1 | Plectin | 137,31 | + |  |  |
|  | P60335 | Poly(rC)-binding protein 1 | 163,18 | -0,053 |  |  |
|  | P29341 | Polyadenylate-binding protein 1 | 285,1 | -0,053 |  |  |
|  | Q8C7U7 | Polypeptide N-acetylgalactosaminyltransferase 6 | 108,8 | - |  |  |
|  | P17225 | Polypyrimidine tract-binding protein 1 | 64,65 | -0,053 |  |  |
|  | P0CG49 | Polyubiquitin-B | 775,53 | -0,053 |  |  |
|  | Q61838 | Pregnancy zone protein | 535,56 | -0,053 |  |  |
|  | Q4FK66 | Pre-mRNA-splicing factor 38A | 138,63 | + |  |  |
|  | Q6NSR8 | Probable aminopeptidase NPEPL1 | 68,43 | - |  |  |
|  | Q8BGV0 | Probable asparagine--tRNA ligase, mitochondrial | 592,28 | - |  |  |
|  | Q61656 | Probable ATP-dependent RNA helicase DDX5 | 235,37 | - |  |  |
|  | Q6PAV2 | Probable E3 ubiquitin-protein ligase HERC4 | 74,4 | + |  |  |
|  | Q8CFI5 | Probable proline--tRNA ligase, mitochondrial | 299,25 | + |  |  |
|  | P01132 | Pro-epidermal growth factor | 75,93 | + |  |  |
|  | P62962 | Profilin-1 | 2726,85 | -0,053 |  |  |
|  | Q8C5N5 | Programmed cell death protein 2-like | 162,38 | + |  |  |
|  | Q61823 | Programmed cell death protein 4 | 98,78 | -0,053 |  |  |
|  | P02816 | Prolactin-inducible protein homolog | 1209,71 | -0,053 |  |  |
|  | E9PVX6 | Proliferation marker protein Ki-67 | 139,98 | -0,053 |  |  |
|  | Q3UUY6 | Prominin-2 | 97,47 | - |  |  |
|  | Q91ZA3 | Propionyl-CoA carboxylase alpha chain, mitochondrial | 331,81 | 1,051 |  |  |
|  | Q80W65 | Proprotein convertase subtilisin/kexin type 9 | 205,31 | - |  |  |
|  | Q9QUM9 | Proteasome subunit alpha type-6 | 134,98 | + |  |  |
|  | Q8CJG1 | Protein argonaute-1 | 101,3 | + |  |  |
|  | Q8CJF8 | Protein argonaute-4 | 153,81 | + |  |  |
|  | Q924A2 | Protein capicua homolog | 56,43 | - |  |  |
|  | D3Z6P0 | Protein disulfide-isomerase A2 | 163,29 | - |  |  |
|  | P27773 | Protein disulfide-isomerase A3 | 255,82 | 1,051 |  |  |
|  | Q8CJF7 | Protein ELYS | 91,11 | + |  |  |
|  | Q9D0F3 | Protein ERGIC-53 | 74,83 | 1,051 |  |  |
|  | Q9DB52 | Protein FAM122A | 117,84 | + |  |  |
|  | Q8CGI1 | Protein FAM193A | 145,68 | + |  |  |
|  | Q3UY90 | Protein FAM198A | 136,29 | + |  |  |
|  | Q8BR27 | Protein FAM214B | 873,31 | - |  |  |
|  | Q8C9E8 | Protein FAM26F | 121,54 | - |  |  |
|  | Q8CG73 | Protein fantom | 1070,01 | + |  |  |
|  | Q99JB8 | Protein kinase C and casein kinase II substrate protein 3 | 240,56 | - |  |  |
|  | Q02111 | Protein kinase C theta type | 89,66 | - |  |  |
|  | Q8CGC4 | Protein LSM14 homolog B | 128,99 | + |  |  |
|  | B1AUR6 | Protein MMS22-like | 336,97 | - |  |  |
|  | O35595 | Protein patched homolog 2 | 141,87 | - |  |  |
|  | Q811G0 | Protein PTHB1 | 236,65 | + |  |  |
|  | P56565 | Protein S100-A1 | 540,71 | + |  |  |
|  | Q8CA71 | Protein shisa-4 | 340,84 | - |  |  |
|  | Q9WTP2 | Protein sprouty homolog 4 | 115,06 | + |  |  |
|  | Q7TNB8 | Protein strawberry notch homolog 2 | 110,63 | + |  |  |
|  | E9PV87 | Protein TALPID3 | 42,04 | + |  |  |
|  | Q01405 | Protein transport protein Sec23A | 204,42 | + |  |  |
|  | Q9D662 | Protein transport protein Sec23B | 204,21 | -0,053 |  |  |
|  | Q8R121 | Protein Z-dependent protease inhibitor | 84,98 | - |  |  |
|  | Q8K3V4 | Protein-arginine deiminase type-6 | 187,56 | - |  |  |
|  | Q91Y13 | Protocadherin alpha-7 | 65,97 | + |  |  |
|  | P16381 | Putative ATP-dependent RNA helicase Pl10 | 99,7 | + |  |  |
|  | O88851 | Putative hydrolase RBBP9 | 134,95 | + |  |  |
|  | Q922W5 | Pyrroline-5-carboxylate reductase 1, mitochondrial | 213,89 | 1,051 |  |  |
|  | Q922Q4 | Pyrroline-5-carboxylate reductase 2 | 95,07 | - |  |  |
|  | Q05920 | Pyruvate carboxylase, mitochondrial | 87,37 | -0,053 |  |  |
|  | Q9D051 | Pyruvate dehydrogenase E1 component subunit beta, mitochondrial | 116,6 | - |  |  |
|  | Q61598 | Rab GDP dissociation inhibitor beta | 315,84 | -0,053 |  |  |
|  | O35551 | Rab GTPase-binding effector protein 1 | 97,34 | + |  |  |
|  | Q69ZJ7 | RAB6A-GEF complex partner protein 1 | 172,17 | - |  |  |
|  | Q8VIG3 | Radial spoke head 1 homolog | 96,19 | - |  |  |
|  | A3KGS3 | Ral GTPase-activating protein subunit alpha-2 | 53,42 | - |  |  |
|  | Q61193 | Ral guanine nucleotide dissociation stimulator-like 2 | 164,72 | - |  |  |
|  | Q8K386 | Ras-related protein Rab-15 | 546,62 | 1,051 |  |  |
|  | P53994 | Ras-related protein Rab-2A | 142,56 | + |  |  |
|  | P59279 | Ras-related protein Rab-2B | 212,52 | + |  |  |
|  | Q8CB87 | Ras-related protein Rab-44 | 127,5 | + |  |  |
|  | Q9ESK9 | RB1-inducible coiled-coil protein 1 | 83,97 | + |  |  |
|  | P68040 | Receptor of activated protein C kinase 1 | 537,69 | 1,051 |  |  |
|  | Q05909 | Receptor-type tyrosine-protein phosphatase gamma | 176,93 | - |  |  |
|  | Q64374 | Regucalcin | 208,41 | + |  |  |
|  | Q0VGM9 | Regulator of telomere elongation helicase 1 | 147,39 | + |  |  |
|  | P06281 | Renin-1 | 4723,6 | + |  |  |
|  | P00796 | Renin-2 | 8938,26 | + |  |  |
|  | P24549 | Retinal dehydrogenase 1 | 180,65 | -0,053 |  |  |
|  | Q62148 | Retinal dehydrogenase 2 | 107,38 | - |  |  |
|  | P13405 | Retinoblastoma-associated protein | 389,09 | - |  |  |
|  | Q61599 | Rho GDP-dissociation inhibitor 2 | 314,35 | + |  |  |
|  | Q8K0Q5 | Rho GTPase-activating protein 18 | 75,06 | + |  |  |
|  | A6X8Z5 | Rho GTPase-activating protein 31 | 93,5 | - |  |  |
|  | Q91YM2 | Rho GTPase-activating protein 35 | 95,68 | + |  |  |
|  | Q91X46 | Rho guanine nucleotide exchange factor 3 | 212,74 | - |  |  |
|  | Q9ES28 | Rho guanine nucleotide exchange factor 7 | 70,73 | - |  |  |
|  | Q7TPS0 | Ribosomal protein S6 kinase alpha-6 | 113,37 | + |  |  |
|  | Q9CYH6 | Ribosome biogenesis regulatory protein homolog | 161,67 | + |  |  |
|  | Q99PL5 | Ribosome-binding protein 1 | 76,05 | -0,053 |  |  |
|  | Q3UF64 | RING finger and transmembrane domain-containing protein 2 | 93,42 | - |  |  |
|  | A1L3T7 | RIPOR family member 3 | 113,54 | + |  |  |
|  | Q9D7H3 | RNA 3'-terminal phosphate cyclase | 244,55 | - |  |  |
|  | Q80TE0 | RNA polymerase II-associated protein 1 | 115,27 | + |  |  |
|  | B2RY56 | RNA-binding protein 25 | 69,29 | - |  |  |
|  | Q9CTH6 | rRNA-processing protein FCF1 homolog | 264,27 | - |  |  |
|  | Q9JMD1 | Scm-like with four MBT domains protein 1 | 185,63 | - |  |  |
|  | A8Y5H7 | SEC14-like protein 1 | 128 | 1,051 |  |  |
|  | Q8R0F9 | SEC14-like protein 4 | 230,74 | - |  |  |
|  | Q9JI02 | Secretoglobin family 2B member 20 | 4234,92 | -0,053 |  |  |
|  | Q7M747 | Secretoglobin family 2B member 24 | 137,18 | -0,053 |  |  |
|  | A7XUX6 | Selection and upkeep of intraepithelial T-cells protein 2 | 141,67 | + |  |  |
|  | P70274 | Selenoprotein P | 142,45 | - |  |  |
|  | O08665 | Semaphorin-3A | 641,32 | - |  |  |
|  | Q62177 | Semaphorin-3B | 107,98 | - |  |  |
|  | P29621 | Serine protease inhibitor A3C | 128,92 | -0,053 |  |  |
|  | Q80X76 | Serine protease inhibitor A3F | 329,09 | -0,053 |  |  |
|  | P07759 | Serine protease inhibitor A3K | 209,73 | -0,053 |  |  |
|  | Q03734 | Serine protease inhibitor A3M | 134,44 | -0,053 |  |  |
|  | Q8BGW6 | Serine/threonine-protein kinase 32A | 204,17 | - |  |  |
|  | Q05512 | Serine/threonine-protein kinase MARK2 | 103,95 | - |  |  |
|  | Q9R0A5 | Serine/threonine-protein kinase Nek3 | 106,14 | - |  |  |
|  | O54949 | Serine/threonine-protein kinase NLK | 170,03 | - |  |  |
|  | Q9QZS5 | Serine/threonine-protein kinase Sgk2 | 88,45 | + |  |  |
|  | Q3UH66 | Serine/threonine-protein kinase WNK2 | 140,29 | - |  |  |
|  | P63087 | Serine/threonine-protein phosphatase PP1-gamma catalytic subunit | 172,36 | + |  |  |
|  | Q921I1 | Serotransferrin | 1669,72 | -0,053 |  |  |
|  | Q60854 | Serpin B6 | 859,83 | + |  |  |
|  | P07724 | Serum albumin | 532,54 | 1,051 |  |  |
|  | Q62087 | Serum paraoxonase/lactonase 3 | 283,55 | - |  |  |
|  | E9PYH6 | SET domain-containing 1A | 73,24 | - |  |  |
|  | Q99JR1 | Sideroflexin-1 | 87,42 | - |  |  |
|  | Q9D8V7 | Signal peptidase complex catalytic subunit SEC11C | 225,83 | - |  |  |
|  | Q9D958 | Signal peptidase complex subunit 1 | 150,87 | + |  |  |
|  | Q9CYN2 | Signal peptidase complex subunit 2 | 104,47 | - |  |  |
|  | P42230 | Signal transducer and activator of transcription 5A | 134,51 | + |  |  |
|  | G3X9J0 | Signal-induced proliferation-associated 1-like protein 3 | 125,34 | - |  |  |
|  | Q61079 | Single-minded homolog 2 | 116,87 | - |  |  |
|  | Q810B9 | SLIT and NTRK-like protein 3 | 126,18 | - |  |  |
|  | Q8VDN2 | Sodium/potassium-transporting ATPase subunit alpha-1 | 97,22 | + |  |  |
|  | Q8BVL3 | Sorting nexin-17 | 93,27 | + |  |  |
|  | Q3ZT31 | Sorting nexin-25 | 130,03 | + |  |  |
|  | Q8BLY1 | SPARC-related modular calcium-binding protein 1 | 207,48 | + |  |  |
|  | Q8BI29 | Specifically androgen-regulated gene protein | 112,29 | - |  |  |
|  | A0AUV4 | Sperm motility kinase Y | 79,45 | - |  |  |
|  | Q64674 | Spermidine synthase | 92,37 | 1,051 |  |  |
|  | Q9Z1N5 | Spliceosome RNA helicase Ddx39b | 132,94 | + |  |  |
|  | O54781 | SRSF protein kinase 2 | 119,01 | - |  |  |
|  | Q8C4H2 | Sterile alpha motif domain-containing protein 3 | 127,35 | - |  |  |
|  | Q69Z37 | Sterile alpha motif domain-containing protein 9-like | 583,25 | + |  |  |
|  | Q499E5 | Storkhead-box protein 2 | 225,9 | + |  |  |
|  | Q9ESP1 | Stromal cell-derived factor 2-like protein 1 | 391,69 | -0,053 |  |  |
|  | Q61900 | Submaxillary gland androgen-regulated protein 3A | 2236,32 | 1,051 |  |  |
|  | Q9Z2I8 | Succinate--CoA ligase [GDP-forming] subunit beta, mitochondrial | 359,71 | + |  |  |
|  | Q8K4L3 | Supervillin | 139,96 | - |  |  |
|  | Q8CH09 | SURP and G-patch domain-containing protein 2 | 130,77 | - |  |  |
|  | Q62465 | Synaptic vesicle membrane protein VAT-1 homolog | 194,14 | + |  |  |
|  | Q62209 | Synaptonemal complex protein 1 | 167,26 | - |  |  |
|  | O35681 | Synaptotagmin-3 | 128,37 | + |  |  |
|  | Q99N80 | Synaptotagmin-like protein 1 | 141,4 | - |  |  |
|  | Q70IV5 | Synemin | 105,32 | - |  |  |
|  | Q5SV85 | Synergin gamma | 78,74 | + |  |  |
|  | Q71LX4 | Talin-2 | 116,17 | - |  |  |
|  | Q6PFX9 | Tankyrase-1 | 108,7 | + |  |  |
|  | Q3UES3 | Tankyrase-2 | 190,09 | - |  |  |
|  | P06335 | T-cell receptor gamma chain C region 5/10-13 | 61,68 | + |  |  |
|  | Q8VC51 | Telomerase Cajal body protein 1 | 135,57 | - |  |  |
|  | D3YTS9 | Testicular acid phosphatase | 165,46 | + |  |  |
|  | Q3URQ0 | Testis-expressed protein 10 | 104,79 | - |  |  |
|  | Q9D6K7 | Tetratricopeptide repeat protein 33 | 98,67 | - |  |  |
|  | P10639 | Thioredoxin | 424,2 | + |  |  |
|  | Q715T0 | Thioredoxin domain-containing protein 3 | 69,3 | - |  |  |
|  | P20108 | Thioredoxin-dependent peroxide reductase, mitochondrial | 691,04 | -0,053 |  |  |
|  | Q3UQ84 | Threonine--tRNA ligase, mitochondrial | 230,41 | - |  |  |
|  | P97770 | THUMP domain-containing protein 3 | 99,02 | - |  |  |
|  | B2RXC1 | Trafficking protein particle complex subunit 11 | 60,89 | - |  |  |
|  | Q93092 | Transaldolase | 165,87 | 1,051 |  |  |
|  | P52955 | Transcription factor LBX1 | 93,83 | - |  |  |
|  | Q571C7 | Transcription factor TFIIIB component B'' homolog | 160,01 | - |  |  |
|  | Q62318 | Transcription intermediary factor 1-beta | 85,11 | + |  |  |
|  | Q8BKY8 | Transcription termination factor 2, mitochondrial | 163,28 | + |  |  |
|  | P37804 | Transgelin | 380,61 | + |  |  |
|  | P40142 | Transketolase | 157,51 | 1,051 |  |  |
|  | P63028 | Translationally-controlled tumor protein | 292,48 | 1,051 |  |  |
|  | Q62186 | Translocon-associated protein subunit delta | 193,7 | - |  |  |
|  | Q7TN60 | Transmembrane channel-like protein 6 | 1418,53 | + |  |  |
|  | Q78IS1 | Transmembrane emp24 domain-containing protein 3 | 220,37 | - |  |  |
|  | Q8R1V4 | Transmembrane emp24 domain-containing protein 4 | 293,39 | -0,053 |  |  |
|  | Q99KF1 | Transmembrane emp24 domain-containing protein 9 | 328,08 | - |  |  |
|  | A7E1Z1 | Transmembrane protein 215 | 113,67 | - |  |  |
|  | P07309 | Transthyretin | 189,04 | -0,053 |  |  |
|  | P17751 | Triosephosphate isomerase | 242,48 | -0,053 |  |  |
|  | Q99PN3 | Tripartite motif-containing protein 26 | 96,61 | + |  |  |
|  | Q99LF4 | tRNA-splicing ligase RtcB homolog | 183,39 | - |  |  |
|  | Q9JKK7 | Tropomodulin-2 | 266,75 | + |  |  |
|  | Q9JHJ0 | Tropomodulin-3 | 139,22 | - |  |  |
|  | P58771 | Tropomyosin alpha-1 chain | 182,28 | - |  |  |
|  | P58774 | Tropomyosin beta chain | 274,72 | - |  |  |
|  | P20801 | Troponin C, skeletal muscle | 125,37 | - |  |  |
|  | P32921 | Tryptophan--tRNA ligase, cytoplasmic | 85,24 | - |  |  |
|  | P05213 | Tubulin alpha-1B chain | 63,07 | 1,051 |  |  |
|  | P68368 | Tubulin alpha-4A chain | 1205,75 | -0,053 |  |  |
|  | Q9JJZ2 | Tubulin alpha-8 chain | 570,26 | -0,053 |  |  |
|  | Q7TMM9 | Tubulin beta-2A chain | 105,68 | -0,053 |  |  |
|  | Q9CWF2 | Tubulin beta-2B chain | 118,54 | -0,053 |  |  |
|  | Q9ERD7 | Tubulin beta-3 chain | 76,85 | -0,053 |  |  |
|  | Q9D6F9 | Tubulin beta-4A chain | 117,03 | -0,053 |  |  |
|  | P68372 | Tubulin beta-4B chain | 117,03 | -0,053 |  |  |
|  | P99024 | Tubulin beta-5 chain | 93,04 | -0,053 |  |  |
|  | Q922F4 | Tubulin beta-6 chain | 76,85 | -0,053 |  |  |
|  | Q60769 | Tumor necrosis factor alpha-induced protein 3 | 291,59 | + |  |  |
|  | P24529 | Tyrosine 3-monooxygenase | 204,65 | - |  |  |
|  | P00520 | Tyrosine-protein kinase ABL1 | 109,33 | - |  |  |
|  | Q62120 | Tyrosine-protein kinase JAK2 | 71,95 | - |  |  |
|  | Q8BUM3 | Tyrosine-protein phosphatase non-receptor type 7 | 137,99 | + |  |  |
|  | P97797 | Tyrosine-protein phosphatase non-receptor type substrate 1 | 257,49 | -0,053 |  |  |
|  | Q640M1 | U3 small nucleolar RNA-associated protein 14 homolog A | 103,68 | + |  |  |
|  | Q99NB8 | Ubiquilin-4 | 246,97 | + |  |  |
|  | P56399 | Ubiquitin carboxyl-terminal hydrolase 5 | 141,46 | + |  |  |
|  | Q6A4J8 | Ubiquitin carboxyl-terminal hydrolase 7 | 89,44 | + |  |  |
|  | Q9R0P9 | Ubiquitin carboxyl-terminal hydrolase isozyme L1 | 80,04 | - |  |  |
|  | P62983 | Ubiquitin-40S ribosomal protein S27a | 775,53 | -0,053 |  |  |
|  | Q02053 | Ubiquitin-like modifier-activating enzyme 1 | 138,93 | -0,053 |  |  |
|  | Q8VDX6 | UDP-D-xylose:ribitol-5-phosphate beta1,4-xylosyltransferase | 98 | + |  |  |
|  | Q6P5E4 | UDP-glucose:glycoprotein glucosyltransferase 1 | 150,22 | -0,053 |  |  |
|  | Q91YN5 | UDP-N-acetylhexosamine pyrophosphorylase | 140,9 | -0,053 |  |  |
|  | E9QMW4 | Uncharacterized protein C16orf96 homolog | 209,04 | - |  |  |
|  | P46735 | Unconventional myosin-Ib | 144,19 | + |  |  |
|  | P58686 | UPF0184 protein C9orf16 homolog | 344,37 | + |  |  |
|  | Q5NCI0 | Up-regulator of cell proliferation | 73,53 | + |  |  |
|  | Q99PM9 | Uridine-cytidine kinase 2 | 92,86 | + |  |  |
|  | Q2QI47 | Usherin | 93,32 | - |  |  |
|  | Q8C0E2 | Vacuolar protein sorting-associated protein 26B | 118,46 | - |  |  |
|  | Q9Z1Q9 | Valine--tRNA ligase | 421,64 | - |  |  |
|  | P20152 | Vimentin | 237,38 | 1,051 |  |  |
|  | P33587 | Vitamin K-dependent protein C | 95,69 | + |  |  |
|  | Q60932 | Voltage-dependent anion-selective channel protein 1 | 185,84 | + |  |  |
|  | P50516 | V-type proton ATPase catalytic subunit A | 269,16 | + |  |  |
|  | Q8K1X1 | WD repeat-containing protein 11 | 198,92 | + |  |  |
|  | Q3U3T8 | WD repeat-containing protein 62 | 105,3 | + |  |  |
|  | P23475 | X-ray repair cross-complementing protein 6 | 197,2 | + |  |  |
|  | Q0P5X5 | Zinc finger B-box domain-containing protein 1 | 141,27 | - |  |  |
|  | E9Q784 | Zinc finger CCCH domain-containing protein 13 | 81,57 | 1,051 |  |  |
|  | Q68FE8 | Zinc finger protein 280D | 111,09 | + |  |  |
|  | Q6NXK2 | Zinc finger protein 532 | 130,23 | + |  |  |
|  | Q61464 | Zinc finger protein 638 | 117,91 | + |  |  |
|  | Q0VGT2 | Zinc finger protein GLI2 | 130,47 | - |  |  |
|  | Q9QX66 | Zinc finger protein neuro-d4 | 149,97 | - |  |  |
|  | Q925H1 | Zinc finger transcription factor Trps1 | 62,59 | + |  |  |
|  | Q60738 | Zinc transporter 1 | 119,22 | - |  |  |
|  | Q6X786 | Zona pellucida-binding protein 2 | 187 | + |  |  |

^a^ The protein accession number was provided by uniprot.org database (<http://www.uniprot.org/>). The identified proteins are organized according to the alphabetical order. Relative differential expression is indicated by positive value, when the protein is upregulated, and by negative values (−), when the protein is downregulated in the comparison between groups. The representation with only a sign (-) or (+) indicates unique expression in the control group (-) or in the fluoride group (+).
