# Supplementary material for: Effects of long-term fluoride exposure are associated with oxidative biochemistry impairment and global proteomic modulation, but not genotoxicity, in parotid glands of mice
Source: PLoS One. 2022 Jan 27;17(1):e0261252. doi: 10.1371/journal.pone.0261252 (PMC8794182; doi:10.1371/journal.pone.0261252)
Supplement: S2 Table — (DOCX) [file pone.0261252.s002.docx]

| **Table 2**. Global proteomic profile of mice parotid glands exposed to 50 mgF/L in comparison to the control group. List of proteins with differential regulation and exclusive expression in both groups. | | | | | | |
| --- | --- | --- | --- | --- | --- | --- |
|  |  |  | **Fold Change** | |  |  |
|  | ***^a^*Access Number** | **Protein name description** | **PLGS Score** | **50mgF/L** |  |  |
|  | Q8VCR2 | 17-beta-hydroxysteroid dehydrogenase 13 | 63,78 | + |  |  |
|  | Q8VDM4 | 26S proteasome non-ATPase regulatory subunit 2 | 64,74 | + |  |  |
|  | P14685 | 26S proteasome non-ATPase regulatory subunit 3 | 66,74 | - |  |  |
|  | P62196 | 26S proteasome regulatory subunit 8 | 75,64 | - |  |  |
|  | Q61733 | 28S ribosomal protein S31, mitochondrial | 118,76 | - |  |  |
|  | P52760 | 2-iminobutanoate/2-iminopropanoate deaminase | 236,08 | + |  |  |
|  | Q9CQ40 | 39S ribosomal protein L49, mitochondrial | 251,83 | - |  |  |
|  | O08756 | 3-hydroxyacyl-CoA dehydrogenase type-2 | 166,36 | 1,116 |  |  |
|  | Q8QZS1 | 3-hydroxyisobutyryl-CoA hydrolase, mitochondrial | 156,51 | - |  |  |
|  | Q8VCH0 | 3-ketoacyl-CoA thiolase B, peroxisomal | 114,12 | -0,896 |  |  |
|  | P63323 | 40S ribosomal protein S12 | 243,87 | -0,896 |  |  |
|  | P62843 | 40S ribosomal protein S15 | 789,57 | - |  |  |
|  | P14131 | 40S ribosomal protein S16 | 421,37 | -0,896 |  |  |
|  | P60867 | 40S ribosomal protein S20 | 221,78 | + |  |  |
|  | P62849 | 40S ribosomal protein S24 | 208,7 | + |  |  |
|  | P97351 | 40S ribosomal protein S3a | 350,31 | -0,896 |  |  |
|  | P97461 | 40S ribosomal protein S5 | 178,37 | + |  |  |
|  | P62242 | 40S ribosomal protein S8 | 122,1 | - |  |  |
|  | P10852 | 4F2 cell-surface antigen heavy chain | 265,35 | - |  |  |
|  | P70290 | 55 kDa erythrocyte membrane protein | 308,48 | - |  |  |
|  | P99027 | 60S acidic ribosomal protein P2 | 1189,82 | -0,896 |  |  |
|  | Q6ZWV3 | 60S ribosomal protein L10 | 169,22 | - |  |  |
|  | P86048 | 60S ribosomal protein L10-like | 169,22 | - |  |  |
|  | P67984 | 60S ribosomal protein L22 | 751,95 | -0,896 |  |  |
|  | P62830 | 60S ribosomal protein L23 | 1000,17 | - |  |  |
|  | P47962 | 60S ribosomal protein L5 | 1599,18 | -0,896 |  |  |
|  | Q9D8M4 | 60S ribosomal protein L7-like 1 | 255,81 | + |  |  |
|  | Q9DCD0 | 6-phosphogluconate dehydrogenase, decarboxylating | 165,43 | + |  |  |
|  | P20029 | 78 kDa glucose-regulated protein | 118,66 | 1,116 |  |  |
|  | Q8CBW3 | Abl interactor 1 | 68,37 | - |  |  |
|  | Q5SWU9 | Acetyl-CoA carboxylase 1 | 213,79 | + |  |  |
|  | Q91XA9 | Acidic mammalian chitinase | 4212,14 | -0,896 |  |  |
|  | Q99KI0 | Aconitate hydratase, mitochondrial | 219,08 | -0,896 |  |  |
|  | Q80YS6 | Actin filament-associated protein 1 | 106,6 | - |  |  |
|  | P68033 | Actin, alpha cardiac muscle 1 | 809,78 | -0,896 |  |  |
|  | P62737 | Actin, aortic smooth muscle | 809,78 | -0,896 |  |  |
|  | Q8CG27 | Actin-like protein 9 | 138,37 | + |  |  |
|  | Q99JY9 | Actin-related protein 3 | 960,67 | + |  |  |
|  | O54967 | Activated CDC42 kinase 1 | 133,62 | + |  |  |
|  | Q8JZN5 | Acyl-CoA dehydrogenase family member 9, mitochondrial | 153,3 | - |  |  |
|  | P31786 | Acyl-CoA-binding protein | 652,18 | + |  |  |
|  | Q8C0T9 | Adenylate cyclase type 10 | 120,25 | + |  |  |
|  | Q3UHD1 | Adhesion G protein-coupled receptor B1 | 58,63 | + |  |  |
|  | Q8CJ12 | Adhesion G-protein coupled receptor G2 | 91,51 | + |  |  |
|  | Q8QZR5 | Alanine aminotransferase 1 | 54,12 | + |  |  |
|  | P00329 | Alcohol dehydrogenase 1 | 233,11 | -0,896 |  |  |
|  | P47739 | Aldehyde dehydrogenase, dimeric NADP-preferring | 117,27 | - |  |  |
|  | Q5SGK3 | Aldehyde oxidase 2 | 118,13 | - |  |  |
|  | Q8K157 | Aldose 1-epimerase | 427,71 | 1,116 |  |  |
|  | P45376 | Aldose reductase | 194,76 | -0,896 |  |  |
|  | Q812G0 | Alpha-1,3-mannosyl-glycoprotein 4-beta-N-acetylglucosaminyltransferase A | 142,71 | - |  |  |
|  | Q9D306 | Alpha-1,3-mannosyl-glycoprotein 4-beta-N-acetylglucosaminyltransferase C | 171,28 | + |  |  |
|  | P07758 | Alpha-1-antitrypsin 1-1 | 177,1 | + |  |  |
|  | P22599 | Alpha-1-antitrypsin 1-2 | 177,1 | + |  |  |
|  | Q00896 | Alpha-1-antitrypsin 1-3 | 177,1 | + |  |  |
|  | Q00897 | Alpha-1-antitrypsin 1-4 | 52,85 | + |  |  |
|  | P29699 | Alpha-2-HS-glycoprotein | 230,12 | 1,116 |  |  |
|  | Q7TPR4 | Alpha-actinin-1 | 134,38 | + |  |  |
|  | O88990 | Alpha-actinin-3 | 79,57 | + |  |  |
|  | P57780 | Alpha-actinin-4 | 181,88 | + |  |  |
|  | Q9QYC0 | Alpha-adducin | 259,69 | -0,896 |  |  |
|  | Q9DBF1 | Alpha-aminoadipic semialdehyde dehydrogenase | 227,3 | -0,896 |  |  |
|  | Q99K67 | Alpha-aminoadipic semialdehyde synthase, mitochondrial | 79,83 | - |  |  |
|  | P00687 | Alpha-amylase 1 | 290,35 | -0,896 |  |  |
|  | Q9D3D0 | Alpha-tocopherol transfer protein-like | 85,32 | + |  |  |
|  | Q8WTY4 | Anamorsin | 193,06 | - |  |  |
|  | P07356 | Annexin A2 | 367,64 | + |  |  |
|  | O88312 | Anterior gradient protein 2 homolog | 222,29 | 1,116 |  |  |
|  | Q8CG79 | Apoptosis-stimulating of p53 protein 2 | 77,34 | - |  |  |
|  | Q8BP47 | Asparagine--tRNA ligase, cytoplasmic | 116,74 | + |  |  |
|  | Q9Z2W0 | Aspartyl aminopeptidase | 270,96 | + |  |  |
|  | Q03265 | ATP synthase subunit alpha, mitochondrial | 805,86 | -0,896 |  |  |
|  | P56480 | ATP synthase subunit beta, mitochondrial | 73,46 | -0,896 |  |  |
|  | Q9DCX2 | ATP synthase subunit d, mitochondrial | 266,79 | + |  |  |
|  | Q9DB20 | ATP synthase subunit O, mitochondrial | 641,36 | - |  |  |
|  | Q91V92 | ATP-citrate synthase | 92,4 | - |  |  |
|  | P47857 | ATP-dependent 6-phosphofructokinase, muscle type | 83,93 | + |  |  |
|  | O70133 | ATP-dependent RNA helicase A | 439,96 | - |  |  |
|  | Q8VDW0 | ATP-dependent RNA helicase DDX39A | 157,32 | + |  |  |
|  | P86174 | BEN domain-containing protein 4 | 104,8 | - |  |  |
|  | Q8BFZ3 | Beta-actin-like protein 2 | 1101,7 | -0,896 |  |  |
|  | Q99MK8 | Beta-adrenergic receptor kinase 1 | 201,92 | - |  |  |
|  | Q9JLV6 | Bifunctional polynucleotide phosphatase/kinase | 663,63 | + |  |  |
|  | P07743 | BPI fold-containing family A member 2 | 801,39 | -0,896 |  |  |
|  | P24288 | Branched-chain-amino-acid aminotransferase, cytosolic | 81,93 | - |  |  |
|  | Q8C3R1 | BRCA1-associated ATM activator 1 | 88,85 | + |  |  |
|  | Q8BMB0 | BRCA2-interacting transcriptional repressor EMSY | 92,12 | - |  |  |
|  | Q3TCJ1 | BRISC complex subunit Abraxas 2 | 129,74 | - |  |  |
|  | Q921C3 | Bromodomain and WD repeat-containing protein 1 | 193,62 | - |  |  |
|  | P70408 | Cadherin-10 | 144,84 | -0,896 |  |  |
|  | P97291 | Cadherin-8 | 175,77 | - |  |  |
|  | Q8BG22 | Calcium-activated chloride channel regulator 2 | 128,37 | - |  |  |
|  | Q08460 | Calcium-activated potassium channel subunit alpha-1 | 45,13 | + |  |  |
|  | Q9QXX4 | Calcium-binding mitochondrial carrier protein Aralar2 | 149,86 | - |  |  |
|  | Q9D6P8 | Calmodulin-like protein 3 | 93,71 | + |  |  |
|  | Q9Z0H8 | CAP-Gly domain-containing linker protein 2 | 109,8 | - |  |  |
|  | Q05A80 | Caprin-2 | 138 | + |  |  |
|  | Q99MZ3 | Carbohydrate-responsive element-binding protein | 195,93 | - |  |  |
|  | P16015 | Carbonic anhydrase 3 | 643,78 | - |  |  |
|  | P48758 | Carbonyl reductase [NADPH] 1 | 371,22 | -0,896 |  |  |
|  | Q8VCT4 | Carboxylesterase 1D | 243,42 | -0,896 |  |  |
|  | Q02248 | Catenin beta-1 | 230,56 | 1,116 |  |  |
|  | Q8CII2 | Cell division cycle protein 123 homolog | 518,28 | - |  |  |
|  | Q8R349 | Cell division cycle protein 16 homolog | 101,44 | - |  |  |
|  | A2AL36 | Centriolin | 67,65 | + |  |  |
|  | Q8CB62 | Centrobin | 166,2 | - |  |  |
|  | Q9CZW2 | Centromere protein N | 219,07 | - |  |  |
|  | Q0VBV7 | Centrosomal protein of 126 kDa | 82,77 | + |  |  |
|  | Q9CZX2 | Centrosomal protein of 89 kDa | 131,8 | - |  |  |
|  | Q60952 | Centrosome-associated protein CEP250 | 90,36 | + |  |  |
|  | A2A6T1 | Cerebellar degeneration-related protein 2-like | 215,81 | - |  |  |
|  | Q5DTK1 | Chondroitin sulfate synthase 3 | 52,43 | + |  |  |
|  | Q8R090 | Chromaffin granule amine transporter | 82,74 | - |  |  |
|  | Q8C6S9 | Cilia- and flagella-associated protein 54 | 209,38 | - |  |  |
|  | Q8CEL2 | Cilia- and flagella-associated protein 61 | 214,99 | - |  |  |
|  | Q80TV8 | CLIP-associating protein 1 | 158,92 | + |  |  |
|  | Q9QZE5 | Coatomer subunit gamma-1 | 172,41 | -0,896 |  |  |
|  | P18760 | Cofilin-1 | 155,25 | - |  |  |
|  | P45591 | Cofilin-2 | 155,25 | - |  |  |
|  | Q8CDV0 | Coiled-coil domain-containing protein 178 | 92,58 | + |  |  |
|  | Q3URS9 | Coiled-coil domain-containing protein 51 | 136,36 | - |  |  |
|  | Q8CDM4 | Coiled-coil domain-containing protein 73 | 179,91 | - |  |  |
|  | Q60847 | Collagen alpha-1(XII) chain | 120,05 | + |  |  |
|  | Q01149 | Collagen alpha-2(I) chain | 44,6 | + |  |  |
|  | Q02788 | Collagen alpha-2(VI) chain | 77,52 | + |  |  |
|  | P06684 | Complement C5 | 63,38 | + |  |  |
|  | Q8K2Z4 | Condensin complex subunit 1 | 340,9 | - |  |  |
|  | Q921L5 | Conserved oligomeric Golgi complex subunit 2 | 149,61 | + |  |  |
|  | Q8R1U1 | Conserved oligomeric Golgi complex subunit 4 | 287,43 | - |  |  |
|  | P68500 | Contactin-5 | 41,49 | + |  |  |
|  | Q9JMB8 | Contactin-6 | 90,78 | + |  |  |
|  | B9EJA2 | Cortactin-binding protein 2 | 174,05 | - |  |  |
|  | P30275 | Creatine kinase U-type, mitochondrial | 118,63 | - |  |  |
|  | Q6ZQ38 | Cullin-associated NEDD8-dissociated protein 1 | 75,46 | - |  |  |
|  | Q9JM84 | Cystatin 10 | 930,37 | -0,896 |  |  |
|  | Q62426 | Cystatin-B | 313,76 | - |  |  |
|  | Q03401 | Cysteine-rich secretory protein 1 | 100,04 | - |  |  |
|  | Q8CGD2 | Cysteine-rich secretory protein LCCL domain-containing 1 | 49,27 | + |  |  |
|  | Q6P8I6 | Cytochrome c oxidase assembly protein COX11, mitochondrial | 367,39 | - |  |  |
|  | P43024 | Cytochrome c oxidase subunit 6A1, mitochondrial | 315,12 | - |  |  |
|  | P56391 | Cytochrome c oxidase subunit 6B1 | 382,04 | + |  |  |
|  | Q91WL5 | Cytochrome P450 4A12A | 121,54 | - |  |  |
|  | P28271 | Cytoplasmic aconitate hydratase | 109,28 | - |  |  |
|  | O88485 | Cytoplasmic dynein 1 intermediate chain 1 | 54,35 | + |  |  |
|  | Q9CPY7 | Cytosol aminopeptidase | 264,55 | -0,896 |  |  |
|  | Q61753 | D-3-phosphoglycerate dehydrogenase | 352,25 | 1,116 |  |  |
|  | Q8K3G9 | DCC-interacting protein 13-beta | 68,43 | - |  |  |
|  | O35215 | D-dopachrome decarboxylase | 630,31 | - |  |  |
|  | O88843 | Death domain-containing protein CRADD | 284,09 | - |  |  |
|  | Q8BIK4 | Dedicator of cytokinesis protein 9 | 83,71 | - |  |  |
|  | Q99KU1 | Dehydrodolichyl diphosphate synthase complex subunit Dhdds | 63,58 | - |  |  |
|  | Q9R0P5 | Destrin | 168,66 | + |  |  |
|  | Q8K4R9 | Disks large-associated protein 5 | 125,32 | - |  |  |
|  | Q9JK91 | DNA mismatch repair protein Mlh1 | 110,06 | + |  |  |
|  | Q6P6J4 | DNA oxidative demethylase ALKBH2 | 970,49 | - |  |  |
|  | Q6PFE3 | DNA repair and recombination protein RAD54B | 216,83 | - |  |  |
|  | Q64511 | DNA topoisomerase 2-beta | 93,69 | - |  |  |
|  | Q6ZQF0 | DNA topoisomerase 2-binding protein 1 | 164,27 | - |  |  |
|  | P52432 | DNA-directed RNA polymerases I and III subunit RPAC1 | 445,95 | - |  |  |
|  | Q99KV1 | DnaJ homolog subfamily B member 11 | 131,4 | -0,896 |  |  |
|  | Q9R022 | DnaJ homolog subfamily C member 12 | 257,48 | - |  |  |
|  | Q91YW3 | DnaJ homolog subfamily C member 3 | 372,09 | -0,896 |  |  |
|  | O54734 | Dolichyl-diphosphooligosaccharide--protein glycosyltransferase 48 kDa subunit | 665,29 | -0,896 |  |  |
|  | Q6XUX1 | Dual serine/threonine and tyrosine protein kinase | 288,08 | - |  |  |
|  | Q8K1M6 | Dynamin-1-like protein | 77,05 | + |  |  |
|  | Q8BZ98 | Dynamin-3 | 108,84 | - |  |  |
|  | Q8CHI8 | E1A-binding protein p400 | 110,66 | 1,116 |  |  |
|  | Q9WVS6 | E3 ubiquitin-protein ligase parkin | 97,14 | - |  |  |
|  | P97868 | E3 ubiquitin-protein ligase RBBP6 | 337,9 | + |  |  |
|  | O35445 | E3 ubiquitin-protein ligase RNF5 | 193,81 | - |  |  |
|  | Q8BGX0 | E3 ubiquitin-protein ligase TRIM23 | 78,35 | + |  |  |
|  | Q6PCX9 | E3 ubiquitin-protein ligase TRIM37 | 138,05 | + |  |  |
|  | Q38HM4 | E3 ubiquitin-protein ligase TRIM63 | 85,14 | - |  |  |
|  | Q8BL66 | Early endosome antigen 1 | 81,99 | - |  |  |
|  | Q99LC5 | Electron transfer flavoprotein subunit alpha, mitochondrial | 165,06 | -0,896 |  |  |
|  | Q9D4C5 | ELL-associated factor 1 | 112,46 | + |  |  |
|  | P57680 | Ellis-van Creveld syndrome protein homolog | 170,42 | - |  |  |
|  | P62631 | Elongation factor 1-alpha 2 | 2869,59 | -0,896 |  |  |
|  | P57776 | Elongation factor 1-delta | 223,24 | -0,896 |  |  |
|  | Q9D8N0 | Elongation factor 1-gamma | 531,83 | -0,896 |  |  |
|  | Q8R311 | Endoplasmic reticulum export factor CTAGE5 | 74,36 | + |  |  |
|  | Q9DC16 | Endoplasmic reticulum-Golgi intermediate compartment protein 1 | 314,28 | - |  |  |
|  | Q8BYG9 | Ephrin type-A receptor 10 | 87,64 | + |  |  |
|  | O35393 | Ephrin-B3 | 141,82 | + |  |  |
|  | Q3US41 | Epithelial splicing regulatory protein 1 | 127,47 | - |  |  |
|  | Q6IE26 | Epoxide hydrolase 4 | 175,66 | -0,896 |  |  |
|  | Q6PH08 | ERC protein 2 | 91,62 | + |  |  |
|  | Q9EQ06 | Estradiol 17-beta-dehydrogenase 11 | 239,52 | + |  |  |
|  | P60843 | Eukaryotic initiation factor 4A-I | 492,27 | -0,896 |  |  |
|  | Q149F3 | Eukaryotic peptide chain release factor GTP-binding subunit ERF3B | 133,5 | + |  |  |
|  | Q3UGC7 | Eukaryotic translation initiation factor 3 subunit J-A | 99,37 | + |  |  |
|  | Q66JS6 | Eukaryotic translation initiation factor 3 subunit J-B | 99,37 | + |  |  |
|  | Q99JX4 | Eukaryotic translation initiation factor 3 subunit M | 138,27 | + |  |  |
|  | P63242 | Eukaryotic translation initiation factor 5A-1 | 174,19 | + |  |  |
|  | Q8BGY2 | Eukaryotic translation initiation factor 5A-2 | 174,19 | + |  |  |
|  | Q3LHH8 | Exocrine gland-secreted peptide 1 | 79,86 | + |  |  |
|  | Q6KAR6 | Exocyst complex component 3 | 43,87 | + |  |  |
|  | Q9CXP9 | Exonuclease V | 293,15 | - |  |  |
|  | Q9JHI7 | Exosome complex component RRP45 | 384,07 | - |  |  |
|  | Q5FW85 | Extracellular matrix protein 2 | 139,56 | + |  |  |
|  | P47753 | F-actin-capping protein subunit alpha-1 | 346,29 | + |  |  |
|  | P47754 | F-actin-capping protein subunit alpha-2 | 181 | + |  |  |
|  | Q9D4K4 | FANCD2 opposite strand protein | 144,01 | - |  |  |
|  | Q920E5 | Farnesyl pyrophosphate synthase | 90,34 | - |  |  |
|  | P47740 | Fatty aldehyde dehydrogenase | 383,43 | -0,896 |  |  |
|  | Q3USJ8 | F-BAR and double SH3 domains protein 2 | 93,82 | - |  |  |
|  | Q9CZV8 | F-box/LRR-repeat protein 20 | 117,44 | + |  |  |
|  | Q3UY23 | Ferredoxin-fold anticodon-binding domain-containing protein 1 homolog | 132,33 | - |  |  |
|  | Q6P6L0 | Filamin A-interacting protein 1-like | 55,54 | + |  |  |
|  | A2AKG8 | Focadhesin | 44,11 | + |  |  |
|  | P05064 | Fructose-bisphosphate aldolase A | 195,34 | -0,896 |  |  |
|  | P05063 | Fructose-bisphosphate aldolase C | 125,78 | + |  |  |
|  | P97807 | Fumarate hydratase, mitochondrial | 215,51 | + |  |  |
|  | Q5RJY2 | G2/M phase-specific E3 ubiquitin-protein ligase | 57,13 | - |  |  |
|  | Q9Z0L8 | Gamma-glutamyl hydrolase | 362,84 | + |  |  |
|  | Q5SSG4 | GAS2-like protein 2 | 239,61 | -0,896 |  |  |
|  | P23591 | GDP-L-fucose synthase | 169,92 | + |  |  |
|  | Q9Z1Z0 | General vesicular transport factor p115 | 183,09 | - |  |  |
|  | P15626 | Glutathione S-transferase Mu 2 | 162,11 | -0,896 |  |  |
|  | Q64467 | Glyceraldehyde-3-phosphate dehydrogenase, testis-specific | 111,45 | -0,896 |  |  |
|  | P13707 | Glycerol-3-phosphate dehydrogenase [NAD(+)], cytoplasmic | 155,36 | + |  |  |
|  | Q61543 | Golgi apparatus protein 1 | 69,93 | + |  |  |
|  | O35166 | Golgi SNAP receptor complex member 2 | 284,76 | - |  |  |
|  | Q8C0K5 | Graves disease carrier protein homolog | 104,36 | - |  |  |
|  | Q60779 | Growth arrest-specific protein 8 | 192,38 | + |  |  |
|  | Q9ESX5 | H/ACA ribonucleoprotein complex subunit 4 | 147,55 | - |  |  |
|  | Q61646 | Haptoglobin | 101,59 | + |  |  |
|  | Q9CZJ2 | Heat shock 70 kDa protein 12B | 52,99 | + |  |  |
|  | Q99NG0 | Helicase ARIP4 | 90,09 | + |  |  |
|  | P01942 | Hemoglobin subunit alpha | 900,76 | -0,896 |  |  |
|  | P02088 | Hemoglobin subunit beta-1 | 2039,06 | -0,896 |  |  |
|  | P02089 | Hemoglobin subunit beta-2 | 1031,21 | -0,896 |  |  |
|  | P06467 | Hemoglobin subunit zeta | 291,89 | 1,116 |  |  |
|  | Q91X72 | Hemopexin | 181,63 | -0,896 |  |  |
|  | Q8BG05 | Heterogeneous nuclear ribonucleoprotein A3 | 105,43 | + |  |  |
|  | P61979 | Heterogeneous nuclear ribonucleoprotein K | 228,48 | + |  |  |
|  | Q8R081 | Heterogeneous nuclear ribonucleoprotein L | 73,93 | + |  |  |
|  | Q9D1P2 | Histone acetyltransferase KAT8 | 90,27 | + |  |  |
|  | Q8C2B3 | Histone deacetylase 7 | 102,76 | + |  |  |
|  | Q8CGP5 | Histone H2A type 1-F | 20828,94 | -0,896 |  |  |
|  | Q8CGP6 | Histone H2A type 1-H | 20828,94 | -0,896 |  |  |
|  | Q8CGP7 | Histone H2A type 1-K | 20828,94 | -0,896 |  |  |
|  | Q6GSS7 | Histone H2A type 2-A | 20828,94 | -0,896 |  |  |
|  | Q64522 | Histone H2A type 2-B | 2548,16 | -0,896 |  |  |
|  | Q64523 | Histone H2A type 2-C | 20828,94 | -0,896 |  |  |
|  | Q8BFU2 | Histone H2A type 3 | 20828,94 | -0,896 |  |  |
|  | Q8R1M2 | Histone H2A.J | 20828,94 | -0,896 |  |  |
|  | Q3THW5 | Histone H2A.V | 2696,72 | -0,896 |  |  |
|  | P0C0S6 | Histone H2A.Z | 2696,72 | -0,896 |  |  |
|  | P27661 | Histone H2AX | 2696,72 | -0,896 |  |  |
|  | P70696 | Histone H2B type 1-A | 3182,92 | -0,896 |  |  |
|  | Q64475 | Histone H2B type 1-B | 10972,62 | -0,896 |  |  |
|  | Q6ZWY9 | Histone H2B type 1-C/E/G | 10972,62 | -0,896 |  |  |
|  | P10853 | Histone H2B type 1-F/J/L | 10972,62 | -0,896 |  |  |
|  | Q64478 | Histone H2B type 1-H | 10972,62 | -0,896 |  |  |
|  | Q8CGP1 | Histone H2B type 1-K | 10972,62 | -0,896 |  |  |
|  | P10854 | Histone H2B type 1-M | 10972,62 | -0,896 |  |  |
|  | Q8CGP2 | Histone H2B type 1-P | 10972,62 | -0,896 |  |  |
|  | Q64525 | Histone H2B type 2-B | 10972,62 | -0,896 |  |  |
|  | Q64524 | Histone H2B type 2-E | 9182,73 | -0,896 |  |  |
|  | Q9D2U9 | Histone H2B type 3-A | 9182,73 | -0,896 |  |  |
|  | Q8CGP0 | Histone H2B type 3-B | 9182,73 | -0,896 |  |  |
|  | P68433 | Histone H3.1 | 865,25 | -0,896 |  |  |
|  | P84228 | Histone H3.2 | 1843,33 | -0,896 |  |  |
|  | P84244 | Histone H3.3 | 1484,99 | -0,896 |  |  |
|  | P02301 | Histone H3.3C | 1484,99 | -0,896 |  |  |
|  | P62806 | Histone H4 | 15321,71 | -0,896 |  |  |
|  | E9Q5F9 | Histone-lysine N-methyltransferase SETD2 | 91,74 | - |  |  |
|  | P97443 | Histone-lysine N-methyltransferase Smyd1 | 103,89 | + |  |  |
|  | P70298 | Homeobox protein cut-like 2 | 139,74 | + |  |  |
|  | O88866 | Hormonally up-regulated neu tumor-associated kinase | 99,05 | + |  |  |
|  | P01878 | Ig alpha chain C region | 822,98 | -0,896 |  |  |
|  | P01868 | Ig gamma-1 chain C region secreted form | 192,37 | + |  |  |
|  | P01869 | Ig gamma-1 chain C region, membrane-bound form | 192,37 | + |  |  |
|  | P01863 | Ig gamma-2A chain C region, A allele | 157,39 | 1,116 |  |  |
|  | P01865 | Ig gamma-2A chain C region, membrane-bound form | 157,39 | 1,116 |  |  |
|  | P01867 | Ig gamma-2B chain C region | 518,82 | -0,896 |  |  |
|  | P01837 | Ig kappa chain C region | 668,32 | - |  |  |
|  | Q8K0C1 | Importin-13 | 86,6 | - |  |  |
|  | Q7M6U3 | Inactive serine/threonine-protein kinase TEX14 | 116,91 | - |  |  |
|  | P15975 | Inactive ubiquitin carboxyl-terminal hydrolase 53 | 182,27 | - |  |  |
|  | P11881 | Inositol 1,4,5-trisphosphate receptor type 1 | 85,51 | + |  |  |
|  | Q9Z0Y7 | Insulin receptor substrate 4 | 54,52 | + |  |  |
|  | Q9Z0R4 | Intersectin-1 | 292,55 | - |  |  |
|  | P15948 | Kallikrein 1-related peptidase b22 | 206,71 | - |  |  |
|  | P36369 | Kallikrein 1-related peptidase b26 | 96,62 | - |  |  |
|  | B1AQ75 | Keratin, type I cuticular Ha6 | 192,32 | -0,896 |  |  |
|  | P05784 | Keratin, type I cytoskeletal 18 | 91,52 | 1,116 |  |  |
|  | A1L317 | Keratin, type I cytoskeletal 24 | 134,19 | -0,896 |  |  |
|  | Q3TRJ4 | Keratin, type I cytoskeletal 26 | 147,98 | - |  |  |
|  | Q99M73 | Keratin, type II cuticular Hb4 | 90,27 | 1,116 |  |  |
|  | P04104 | Keratin, type II cytoskeletal 1 | 129,26 | + |  |  |
|  | Q6IFZ6 | Keratin, type II cytoskeletal 1b | 90,23 | + |  |  |
|  | Q3UV17 | Keratin, type II cytoskeletal 2 oral | 90,27 | -0,896 |  |  |
|  | P50446 | Keratin, type II cytoskeletal 6A | 90,27 | 1,116 |  |  |
|  | Q9Z331 | Keratin, type II cytoskeletal 6B | 90,27 | 1,116 |  |  |
|  | Q6IME9 | Keratin, type II cytoskeletal 72 | 138,68 | + |  |  |
|  | Q8BGZ7 | Keratin, type II cytoskeletal 75 | 191,62 | -0,896 |  |  |
|  | P11679 | Keratin, type II cytoskeletal 8 | 235,39 | -0,896 |  |  |
|  | P33175 | Kinesin heavy chain isoform 5A | 74,25 | - |  |  |
|  | Q99PT9 | Kinesin-like protein KIF19 | 98,61 | + |  |  |
|  | Q71RI9 | Kynurenine--oxoglutarate transaminase 3 | 244 | - |  |  |
|  | Q66VB7 | Lacrein | 614,14 | -0,896 |  |  |
|  | Q99KP3 | Lambda-crystallin homolog | 164,98 | - |  |  |
|  | P02468 | Laminin subunit gamma-1 | 102,44 | - |  |  |
|  | Q80Y17 | Lethal(2) giant larvae protein homolog 1 | 86,9 | - |  |  |
|  | Q3UMG5 | Leucine-rich repeat and calponin homology domain-containing protein 2 | 119,95 | + |  |  |
|  | Q61809 | Leucine-rich repeat neuronal protein 1 | 119,53 | - |  |  |
|  | Q5S006 | Leucine-rich repeat serine/threonine-protein kinase 2 | 98,31 | - |  |  |
|  | Q3UVD5 | Leucine-rich repeat-containing G-protein coupled receptor 6 | 86,88 | - |  |  |
|  | Q148V7 | LisH domain and HEAT repeat-containing protein KIAA1468 | 122,68 | + |  |  |
|  | P06151 | L-lactate dehydrogenase A chain | 249,9 | + |  |  |
|  | P35951 | Low-density lipoprotein receptor | 128,36 | - |  |  |
|  | P51885 | Lumican | 113,48 | 1,116 |  |  |
|  | P32067 | Lupus La protein homolog | 349,19 | + |  |  |
|  | P46737 | Lys-63-specific deubiquitinase BRCC36 | 189,08 | + |  |  |
|  | Q99MN1 | Lysine--tRNA ligase | 113,62 | + |  |  |
|  | P34884 | Macrophage migration inhibitory factor | 421,77 | - |  |  |
|  | P11590 | Major urinary protein 4 | 353,56 | -0,896 |  |  |
|  | Q9EQK5 | Major vault protein | 309,95 | - |  |  |
|  | P08249 | Malate dehydrogenase, mitochondrial | 630,96 | -0,896 |  |  |
|  | Q8R3F5 | Malonyl-CoA-acyl carrier protein transacylase, mitochondrial | 128,42 | - |  |  |
|  | Q0PMG2 | MAM domain-containing glycosylphosphatidylinositol anchor protein 1 | 270,01 | - |  |  |
|  | Q9DB40 | Mediator of RNA polymerase II transcription subunit 27 | 94,94 | - |  |  |
|  | Q91ZV0 | Melanoma inhibitory activity protein 2 | 72,34 | + |  |  |
|  | Q9WVQ1 | Membrane-associated guanylate kinase, WW and PDZ domain-containing protein 2 | 116,08 | - |  |  |
|  | Q9CXI5 | Mesencephalic astrocyte-derived neurotrophic factor | 356,35 | -0,896 |  |  |
|  | P70669 | Metalloendopeptidase homolog PEX | 218,27 | - |  |  |
|  | Q91ZG2 | Metallophosphoesterase domain-containing protein 1 | 207,27 | + |  |  |
|  | A6H5Y3 | Methionine synthase | 41,09 | + |  |  |
|  | Q9D9H3 | Methyl-CpG-binding domain protein 3-like 1 | 137,85 | + |  |  |
|  | Q9EQ20 | Methylmalonate-semialdehyde dehydrogenase [acylating], mitochondrial | 289,52 | -0,896 |  |  |
|  | P48377 | MHC class II regulatory factor RFX1 | 102,97 | + |  |  |
|  | Q9CXT8 | Mitochondrial-processing peptidase subunit beta | 124,12 | + |  |  |
|  | Q920G8 | Mitoferrin-1 | 163,18 | -0,896 |  |  |
|  | P63085 | Mitogen-activated protein kinase 1 | 178,2 | - |  |  |
|  | Q63844 | Mitogen-activated protein kinase 3 | 169,41 | - |  |  |
|  | Q91Y86 | Mitogen-activated protein kinase 8 | 165,67 | -0,896 |  |  |
|  | Q9D071 | MMS19 nucleotide excision repair protein homolog | 155,93 | - |  |  |
|  | P19467 | Mucin-13 | 65,05 | - |  |  |
|  | P09542 | Myosin light chain 3 | 92,27 | + |  |  |
|  | P09541 | Myosin light chain 4 | 188,87 | - |  |  |
|  | Q5SX40 | Myosin-1 | 281,4 | -0,896 |  |  |
|  | P13541 | Myosin-3 | 172,23 | -0,896 |  |  |
|  | Q5SX39 | Myosin-4 | 308,31 | -0,896 |  |  |
|  | Q02566 | Myosin-6 | 236,87 | -0,896 |  |  |
|  | Q91Z83 | Myosin-7 | 234,02 | -0,896 |  |  |
|  | A2AQP0 | Myosin-7B | 155,75 | -0,896 |  |  |
|  | P13542 | Myosin-8 | 296,4 | -0,896 |  |  |
|  | Q9Z2C4 | Myotubularin-related protein 1 | 260,49 | - |  |  |
|  | Q6ZPE2 | Myotubularin-related protein 5 | 210,43 | - |  |  |
|  | P70441 | Na(+)/H(+) exchange regulatory cofactor NHE-RF1 | 147,15 | - |  |  |
|  | Q99MJ6 | Na(+)/H(+) exchange regulatory cofactor NHE-RF4 | 88,77 | - |  |  |
|  | Q8CIB9 | N-acetyltransferase ESCO2 | 155,56 | + |  |  |
|  | Q66X22 | NACHT, LRR and PYD domains-containing protein 9B | 138,91 | - |  |  |
|  | Q9CPP6 | NADH dehydrogenase [ubiquinone] 1 alpha subcomplex subunit 5 | 355,65 | - |  |  |
|  | Q9DCN2 | NADH-cytochrome b5 reductase 3 | 145,44 | - |  |  |
|  | Q80UM3 | N-alpha-acetyltransferase 15, NatA auxiliary subunit | 362,44 | + |  |  |
|  | P19426 | Negative elongation factor E | 195,44 | - |  |  |
|  | P70232 | Neural cell adhesion molecule L1-like protein | 170,28 | - |  |  |
|  | P08553 | Neurofilament medium polypeptide | 62,1 | + |  |  |
|  | O88522 | NF-kappa-B essential modulator | 272,26 | + |  |  |
|  | Q8CAF4 | NHS-like protein 1 | 167,51 | - |  |  |
|  | Q8R1F1 | Niban-like protein 1 | 466,24 | + |  |  |
|  | P29477 | Nitric oxide synthase, inducible | 78,92 | + |  |  |
|  | Q60974 | Nuclear receptor corepressor 1 | 24,07 | - |  |  |
|  | Q8C163 | Nuclease EXOG, mitochondrial | 268,09 | - |  |  |
|  | Q61937 | Nucleophosmin | 227,54 | + |  |  |
|  | P15532 | Nucleoside diphosphate kinase A | 1534,19 | -0,896 |  |  |
|  | Q01768 | Nucleoside diphosphate kinase B | 136,82 | -0,896 |  |  |
|  | O08919 | Numb-like protein | 130,43 | + |  |  |
|  | Q9D3H2 | Odorant-binding protein 1a | 1146,93 | 1,116 |  |  |
|  | Q9D478 | Outer dense fiber protein 2-like | 85,89 | - |  |  |
|  | P00688 | Pancreatic alpha-amylase | 4613,28 | -0,896 |  |  |
|  | P17742 | Peptidyl-prolyl cis-trans isomerase A | 1327,07 | -0,896 |  |  |
|  | P24369 | Peptidyl-prolyl cis-trans isomerase B | 510,45 | -0,896 |  |  |
|  | P51660 | Peroxisomal multifunctional enzyme type 2 | 709,46 | -0,896 |  |  |
|  | Q2PFD7 | PH and SEC7 domain-containing protein 3 | 73,35 | - |  |  |
|  | Q8C0C7 | Phenylalanine--tRNA ligase alpha subunit | 126,12 | - |  |  |
|  | P70296 | Phosphatidylethanolamine-binding protein 1 | 897,26 | -0,896 |  |  |
|  | O70167 | Phosphatidylinositol 4-phosphate 3-kinase C2 domain-containing subunit gamma | 127,09 | - |  |  |
|  | Q9QYT7 | Phosphatidylinositol N-acetylglucosaminyltransferase subunit Q | 362,91 | - |  |  |
|  | P97813 | Phospholipase D2 | 212,82 | + |  |  |
|  | Q3UMZ3 | Phospholipid phosphatase 5 | 214,44 | - |  |  |
|  | Q8BWJ3 | Phosphorylase b kinase regulatory subunit alpha, liver isoform | 96,71 | + |  |  |
|  | P18826 | Phosphorylase b kinase regulatory subunit alpha, skeletal muscle isoform | 95,37 | + |  |  |
|  | Q61233 | Plastin-2 | 150,29 | - |  |  |
|  | Q08481 | Platelet endothelial cell adhesion molecule | 178,28 | + |  |  |
|  | Q9JIY0 | Pleckstrin homology domain-containing family O member 1 | 131,72 | + |  |  |
|  | Q61990 | Poly(rC)-binding protein 2 | 156,27 | + |  |  |
|  | P57722 | Poly(rC)-binding protein 3 | 160,07 | + |  |  |
|  | P29341 | Polyadenylate-binding protein 1 | 285,1 | -0,896 |  |  |
|  | Q8C7U7 | Polypeptide N-acetylgalactosaminyltransferase 6 | 108,8 | - |  |  |
|  | Q91Z31 | Polypyrimidine tract-binding protein 2 | 192,27 | - |  |  |
|  | P0CG49 | Polyubiquitin-B | 775,53 | -0,896 |  |  |
|  | P0CG50 | Polyubiquitin-C | 775,53 | -0,896 |  |  |
|  | P25425 | POU domain, class 2, transcription factor 1 | 247,97 | - |  |  |
|  | P62515 | POU domain, class 3, transcription factor 4 | 326,11 | - |  |  |
|  | Q6NSR8 | Probable aminopeptidase NPEPL1 | 68,43 | - |  |  |
|  | Q8BGV0 | Probable asparagine--tRNA ligase, mitochondrial | 592,28 | - |  |  |
|  | Q5XF90 | Probable cation-transporting ATPase 13A4 | 131,23 | + |  |  |
|  | P86049 | Probable RNA-binding protein 46 | 73,82 | + |  |  |
|  | P70398 | Probable ubiquitin carboxyl-terminal hydrolase FAF-X | 127,76 | + |  |  |
|  | P67778 | Prohibitin | 168,19 | + |  |  |
|  | P02816 | Prolactin-inducible protein homolog | 1209,71 | -0,896 |  |  |
|  | E9PVX6 | Proliferation marker protein Ki-67 | 139,98 | - |  |  |
|  | Q3V1T4 | Prolyl 3-hydroxylase 1 | 97,52 | + |  |  |
|  | Q3UUY6 | Prominin-2 | 97,47 | - |  |  |
|  | Q80W65 | Proprotein convertase subtilisin/kexin type 9 | 205,31 | - |  |  |
|  | Q9R1P0 | Proteasome subunit alpha type-4 | 175,09 | + |  |  |
|  | Q8K1C0 | Protein angel homolog 2 | 492,29 | - |  |  |
|  | Q8C6G1 | Protein C21orf2 homolog | 233,02 | - |  |  |
|  | Q924A2 | Protein capicua homolog | 56,43 | - |  |  |
|  | D3Z6P0 | Protein disulfide-isomerase A2 | 163,29 | - |  |  |
|  | Q922R8 | Protein disulfide-isomerase A6 | 728,52 | -0,896 |  |  |
|  | Q9D0F3 | Protein ERGIC-53 | 74,83 | -0,896 |  |  |
|  | Q8BR27 | Protein FAM214B | 873,31 | - |  |  |
|  | Q8C9E8 | Protein FAM26F | 121,54 | - |  |  |
|  | E9Q8I9 | Protein furry homolog | 120,38 | - |  |  |
|  | Q059U7 | Protein inturned | 41,77 | + |  |  |
|  | Q99JB8 | Protein kinase C and casein kinase II substrate protein 3 | 240,56 | - |  |  |
|  | Q02111 | Protein kinase C theta type | 89,66 | - |  |  |
|  | Q8C6C9 | Protein LEG1 homolog | 10236,88 | -0,896 |  |  |
|  | B1AUR6 | Protein MMS22-like | 336,97 | - |  |  |
|  | O55126 | Protein NipSnap homolog 2 | 79,97 | - |  |  |
|  | C3VPR6 | Protein NLRC5 | 146,06 | - |  |  |
|  | Q9QZS3 | Protein numb homolog | 120,86 | + |  |  |
|  | O35595 | Protein patched homolog 2 | 141,87 | - |  |  |
|  | Q1W617 | Protein Shroom4 | 113,28 | + |  |  |
|  | Q9D7R2 | Protein TMEPAI | 292,01 | + |  |  |
|  | Q01405 | Protein transport protein Sec23A | 119,07 | + |  |  |
|  | Q9D662 | Protein transport protein Sec23B | 204,21 | -0,896 |  |  |
|  | B2RUP2 | Protein unc-13 homolog D | 242,99 | - |  |  |
|  | Q8K3V4 | Protein-arginine deiminase type-6 | 187,56 | - |  |  |
|  | P23492 | Purine nucleoside phosphorylase | 169,13 | + |  |  |
|  | Q922W5 | Pyrroline-5-carboxylate reductase 1, mitochondrial | 213,89 | - |  |  |
|  | Q05920 | Pyruvate carboxylase, mitochondrial | 87,37 | -0,896 |  |  |
|  | Q9D051 | Pyruvate dehydrogenase E1 component subunit beta, mitochondrial | 116,6 | - |  |  |
|  | P53657 | Pyruvate kinase PKLR | 54,56 | + |  |  |
|  | P50396 | Rab GDP dissociation inhibitor alpha | 307,95 | + |  |  |
|  | Q61598 | Rab GDP dissociation inhibitor beta | 315,84 | -0,896 |  |  |
|  | Q69ZJ7 | RAB6A-GEF complex partner protein 1 | 172,17 | - |  |  |
|  | Q8VIG3 | Radial spoke head 1 homolog | 96,19 | - |  |  |
|  | Q61193 | Ral guanine nucleotide dissociation stimulator-like 2 | 164,72 | - |  |  |
|  | Q99NF8 | Ran-binding protein 17 | 88,14 | + |  |  |
|  | P62821 | Ras-related protein Rab-1A | 286,06 | -0,896 |  |  |
|  | Q9ESK9 | RB1-inducible coiled-coil protein 1 | 80,96 | + |  |  |
|  | P68040 | Receptor of activated protein C kinase 1 | 537,69 | -0,896 |  |  |
|  | Q64487 | Receptor-type tyrosine-protein phosphatase delta | 75,11 | - |  |  |
|  | Q05909 | Receptor-type tyrosine-protein phosphatase gamma | 176,93 | - |  |  |
|  | B1AUH1 | Receptor-type tyrosine-protein phosphatase U | 73,55 | + |  |  |
|  | P24549 | Retinal dehydrogenase 1 | 180,65 | - |  |  |
|  | Q62148 | Retinal dehydrogenase 2 | 107,38 | - |  |  |
|  | P13405 | Retinoblastoma-associated protein | 389,09 | - |  |  |
|  | Q61599 | Rho GDP-dissociation inhibitor 2 | 361,89 | + |  |  |
|  | A6X8Z5 | Rho GTPase-activating protein 31 | 93,5 | - |  |  |
|  | Q91YM2 | Rho GTPase-activating protein 35 | 45,03 | + |  |  |
|  | Q91X46 | Rho guanine nucleotide exchange factor 3 | 212,74 | - |  |  |
|  | Q9ES28 | Rho guanine nucleotide exchange factor 7 | 70,73 | - |  |  |
|  | Q99PL5 | Ribosome-binding protein 1 | 76,05 | -0,896 |  |  |
|  | Q99LE1 | RILP-like protein 2 | 169,71 | + |  |  |
|  | Q3UF64 | RING finger and transmembrane domain-containing protein 2 | 93,42 | - |  |  |
|  | Q99MB7 | RING finger protein 141 | 938,05 | - |  |  |
|  | Q9D7H3 | RNA 3'-terminal phosphate cyclase | 244,55 | - |  |  |
|  | B2RY56 | RNA-binding protein 25 | 69,29 | - |  |  |
|  | O89086 | RNA-binding protein 3 | 450,14 | - |  |  |
|  | Q91WT8 | RNA-binding protein 47 | 356,85 | - |  |  |
|  | Q9CTH6 | rRNA-processing protein FCF1 homolog | 264,27 | - |  |  |
|  | Q9JMD1 | Scm-like with four MBT domains protein 1 | 185,63 | - |  |  |
|  | Q8BRF7 | Sec1 family domain-containing protein 1 | 143,18 | + |  |  |
|  | A8Y5H7 | SEC14-like protein 1 | 128 | - |  |  |
|  | Q8R0F9 | SEC14-like protein 4 | 230,74 | -0,896 |  |  |
|  | Q6UGQ3 | Secretoglobin family 2B member 2 | 5872,32 | -0,896 |  |  |
|  | Q9JI02 | Secretoglobin family 2B member 20 | 4234,92 | -0,896 |  |  |
|  | P70274 | Selenoprotein P | 142,45 | - |  |  |
|  | O08665 | Semaphorin-3A | 641,32 | - |  |  |
|  | Q62177 | Semaphorin-3B | 107,98 | - |  |  |
|  | P07759 | Serine protease inhibitor A3K | 209,73 | 1,116 |  |  |
|  | Q8BGW6 | Serine/threonine-protein kinase 32A | 204,17 | - |  |  |
|  | Q05512 | Serine/threonine-protein kinase MARK2 | 103,95 | - |  |  |
|  | Q9R0A5 | Serine/threonine-protein kinase Nek3 | 106,14 | - |  |  |
|  | O54949 | Serine/threonine-protein kinase NLK | 170,03 | - |  |  |
|  | Q3UH66 | Serine/threonine-protein kinase WNK2 | 140,29 | - |  |  |
|  | P07724 | Serum albumin | 532,54 | -0,896 |  |  |
|  | E9PXF8 | SET-binding factor 2 | 226,13 | -0,896 |  |  |
|  | Q8BSD5 | SH2 domain-containing adapter protein E | 93,61 | + |  |  |
|  | P97306 | SH3 and cysteine-rich domain-containing protein | 523,72 | + |  |  |
|  | Q61056 | Short transient receptor potential channel 1 | 69,44 | + |  |  |
|  | Q99J77 | Sialic acid synthase | 230,66 | -0,896 |  |  |
|  | Q99JR1 | Sideroflexin-1 | 87,42 | - |  |  |
|  | Q9CYN2 | Signal peptidase complex subunit 2 | 104,47 | - |  |  |
|  | G3X9J0 | Signal-induced proliferation-associated 1-like protein 3 | 125,34 | - |  |  |
|  | Q8BQM7 | Single-pass membrane and coiled-coil domain-containing protein 3 | 97,96 | + |  |  |
|  | Q810B9 | SLIT and NTRK-like protein 3 | 126,18 | - |  |  |
|  | P55012 | Solute carrier family 12 member 2 | 80,41 | + |  |  |
|  | Q3UTJ2 | Sorbin and SH3 domain-containing protein 2 | 52,25 | + |  |  |
|  | Q9WV80 | Sorting nexin-1 | 152,15 | - |  |  |
|  | Q8BI29 | Specifically androgen-regulated gene protein | 112,29 | - |  |  |
|  | A0AUV4 | Sperm motility kinase Y | 79,45 | - |  |  |
|  | Q80ZX8 | Sperm-associated antigen 1 | 84,95 | + |  |  |
|  | Q9Z1N5 | Spliceosome RNA helicase Ddx39b | 198,71 | + |  |  |
|  | Q78PY7 | Staphylococcal nuclease domain-containing protein 1 | 400,57 | -0,896 |  |  |
|  | Q8C4H2 | Sterile alpha motif domain-containing protein 3 | 127,35 | - |  |  |
|  | Q62407 | Striated muscle-specific serine/threonine-protein kinase | 156,73 | + |  |  |
|  | Q9ESP1 | Stromal cell-derived factor 2-like protein 1 | 391,69 | -0,896 |  |  |
|  | Q61900 | Submaxillary gland androgen-regulated protein 3A | 2236,32 | -0,896 |  |  |
|  | Q8C341 | SUN domain-containing ossification factor | 59,46 | + |  |  |
|  | P08228 | Superoxide dismutase [Cu-Zn] | 2604,79 | -0,896 |  |  |
|  | Q8K4L3 | Supervillin | 139,96 | - |  |  |
|  | Q8CH09 | SURP and G-patch domain-containing protein 2 | 130,77 | - |  |  |
|  | Q9JIS5 | Synaptic vesicle glycoprotein 2A | 48,32 | + |  |  |
|  | Q62465 | Synaptic vesicle membrane protein VAT-1 homolog | 273,58 | + |  |  |
|  | Q62209 | Synaptonemal complex protein 1 | 167,26 | - |  |  |
|  | Q99N80 | Synaptotagmin-like protein 1 | 141,4 | - |  |  |
|  | Q70IV5 | Synemin | 105,32 | -0,896 |  |  |
|  | Q71LX4 | Talin-2 | 116,17 | - |  |  |
|  | Q3UES3 | Tankyrase-2 | 190,09 | - |  |  |
|  | Q8BKH7 | Target of rapamycin complex 2 subunit MAPKAP1 | 89,56 | + |  |  |
|  | Q8CGA2 | TBC1 domain family member 14 | 92,19 | + |  |  |
|  | Q8VC51 | Telomerase Cajal body protein 1 | 135,57 | - |  |  |
|  | Q1H9T6 | Telomere zinc finger-associated protein | 195,18 | + |  |  |
|  | Q3URQ0 | Testis-expressed protein 10 | 104,79 | - |  |  |
|  | Q14AT2 | Testis-expressed protein 11 | 56,48 | + |  |  |
|  | Q925K9 | Testis-specific serine/threonine-protein kinase 6 | 70,22 | + |  |  |
|  | Q9D6K7 | Tetratricopeptide repeat protein 33 | 98,67 | - |  |  |
|  | Q8C0Q3 | Tetratricopeptide repeat protein 34 | 118,04 | + |  |  |
|  | A3KMP2 | Tetratricopeptide repeat protein 38 | 78,8 | + |  |  |
|  | P10639 | Thioredoxin | 375,48 | + |  |  |
|  | Q715T0 | Thioredoxin domain-containing protein 3 | 69,3 | - |  |  |
|  | Q91W90 | Thioredoxin domain-containing protein 5 | 81,86 | -0,896 |  |  |
|  | P20108 | Thioredoxin-dependent peroxide reductase, mitochondrial | 691,04 | -0,896 |  |  |
|  | Q8BKT7 | THO complex subunit 5 homolog | 97,35 | + |  |  |
|  | Q3UQ84 | Threonine--tRNA ligase, mitochondrial | 230,41 | - |  |  |
|  | Q9WUU8 | TNFAIP3-interacting protein 1 | 75,35 | + |  |  |
|  | B2RXC1 | Trafficking protein particle complex subunit 11 | 60,89 | - |  |  |
|  | Q61286 | Transcription factor 12 | 123,93 | + |  |  |
|  | P52955 | Transcription factor LBX1 | 93,83 | - |  |  |
|  | Q571C7 | Transcription factor TFIIIB component B'' homolog | 160,01 | - |  |  |
|  | Q9JJ11 | Transforming acidic coiled-coil-containing protein 3 | 255,2 | + |  |  |
|  | Q01853 | Transitional endoplasmic reticulum ATPase | 96,41 | -0,896 |  |  |
|  | P63028 | Translationally-controlled tumor protein | 292,48 | 1,116 |  |  |
|  | Q9CY50 | Translocon-associated protein subunit alpha | 90 | + |  |  |
|  | Q62186 | Translocon-associated protein subunit delta | 193,7 | - |  |  |
|  | Q7TN60 | Transmembrane channel-like protein 6 | 777,07 | + |  |  |
|  | Q78IS1 | Transmembrane emp24 domain-containing protein 3 | 220,37 | - |  |  |
|  | A7E1Z1 | Transmembrane protein 215 | 113,67 | - |  |  |
|  | Q8BMS1 | Trifunctional enzyme subunit alpha, mitochondrial | 82,83 | -0,896 |  |  |
|  | Q99JY0 | Trifunctional enzyme subunit beta, mitochondrial | 111,92 | - |  |  |
|  | Q99LF4 | tRNA-splicing ligase RtcB homolog | 183,39 | - |  |  |
|  | Q9JHJ0 | Tropomodulin-3 | 139,22 | - |  |  |
|  | P58771 | Tropomyosin alpha-1 chain | 182,28 | - |  |  |
|  | P58774 | Tropomyosin beta chain | 274,72 | - |  |  |
|  | P20801 | Troponin C, skeletal muscle | 125,37 | - |  |  |
|  | P32921 | Tryptophan--tRNA ligase, cytoplasmic | 85,24 | - |  |  |
|  | Q3UX10 | Tubulin alpha chain-like 3 | 48,92 | + |  |  |
|  | P05213 | Tubulin alpha-1B chain | 63,07 | -0,896 |  |  |
|  | Q9ERD7 | Tubulin beta-3 chain | 76,85 | -0,896 |  |  |
|  | Q9D6F9 | Tubulin beta-4A chain | 117,03 | -0,896 |  |  |
|  | P68372 | Tubulin beta-4B chain | 117,03 | -0,896 |  |  |
|  | P99024 | Tubulin beta-5 chain | 93,04 | -0,896 |  |  |
|  | Q922F4 | Tubulin beta-6 chain | 76,85 | -0,896 |  |  |
|  | Q61333 | Tumor necrosis factor alpha-induced protein 2 | 149,61 | + |  |  |
|  | P24529 | Tyrosine 3-monooxygenase | 204,65 | - |  |  |
|  | P00520 | Tyrosine-protein kinase ABL1 | 109,33 | - |  |  |
|  | Q62120 | Tyrosine-protein kinase JAK2 | 71,95 | - |  |  |
|  | E0CYM8 | Tyrosine-protein phosphatase non-receptor type substrate 1 | 131,04 | -0,896 |  |  |
|  | Q91WQ3 | Tyrosine--tRNA ligase, cytoplasmic | 93,11 | + |  |  |
|  | Q3V0C5 | Ubiquitin carboxyl-terminal hydrolase 48 | 86,4 | + |  |  |
|  | Q9R0P9 | Ubiquitin carboxyl-terminal hydrolase isozyme L1 | 80,04 | - |  |  |
|  | P62983 | Ubiquitin-40S ribosomal protein S27a | 775,53 | -0,896 |  |  |
|  | Q8VE47 | Ubiquitin-like modifier-activating enzyme 5 | 62,99 | + |  |  |
|  | Q6P5E4 | UDP-glucose:glycoprotein glucosyltransferase 1 | 150,22 | -0,896 |  |  |
|  | Q8CGY8 | UDP-N-acetylglucosamine--peptide N-acetylglucosaminyltransferase 110 kDa subunit | 81,95 | + |  |  |
|  | Q9D3J9 | Uncharacterized protein CXorf21 homolog | 93 | + |  |  |
|  | Q2QI47 | Usherin | 93,32 | - |  |  |
|  | Q8C0E2 | Vacuolar protein sorting-associated protein 26B | 118,46 | - |  |  |
|  | Q0P5W1 | Vacuolar protein sorting-associated protein 8 homolog | 86,09 | + |  |  |
|  | Q8VDJ3 | Vigilin | 166,51 | -0,896 |  |  |
|  | Q9ERF3 | WD repeat-containing protein 61 | 142,72 | + |  |  |
|  | Q0P5X5 | Zinc finger B-box domain-containing protein 1 | 141,27 | - |  |  |
|  | E9Q784 | Zinc finger CCCH domain-containing protein 13 | 81,57 | - |  |  |
|  | Q8JZL0 | Zinc finger protein 467 | 203,12 | + |  |  |
|  | Q0VGT2 | Zinc finger protein GLI2 | 130,47 | - |  |  |
|  | Q9QX66 | Zinc finger protein neuro-d4 | 149,97 | - |  |  |
|  | Q60738 | Zinc transporter 1 | 119,22 | - |  |  |

^a^ The protein accession number was provided by uniprot.org database (<http://www.uniprot.org/>). The identified proteins are organized according to the alphabetical order. Relative differential expression is indicated by positive value, when the protein is upregulated, and by negative values (−), when the protein is downregulated in the comparison between groups. The representation with only a sign (-) or (+) indicates unique expression in the control group (-) or in the fluoride group (+).
