# Supplementary material for: Effects of long-term fluoride exposure are associated with oxidative biochemistry impairment and global proteomic modulation, but not genotoxicity, in parotid glands of mice
Source: PLoS One. 2022 Jan 27;17(1):e0261252. doi: 10.1371/journal.pone.0261252 (PMC8794182; doi:10.1371/journal.pone.0261252)
Supplement: S3 Table — (DOCX) [file pone.0261252.s003.docx]

**Supplementary table 3.** Proteins identified according Over-Representation Analysis (ORA) in mice parotid gland, after 60 days of fluoride exposure.

| **^a^Access number** | **Protein name description** | **T10 x C** | **T50 x C** |
| --- | --- | --- | --- |
| B2RWS6 | Histone acetyltransferase p300 | upregulated | - |
| E9Q5F9 | Histone-lysine N-methyltransferase SETD2 | downregulated | downregulated |
| O70456 | 14-3-3 protein sigma | downregulated | - |
| O88569 | Heterogeneous nuclear ribonucleoproteins A2/B1 | upregulated | - |
| O88990 | Alpha-actinin-3 | - | upregulated |
| P00520 | Tyrosine-protein kinase ABL1 | downregulated | downregulated |
| P05213 | Tubulin alpha-1B chain | upregulated | downregulated |
| P0C0S6 | Histone H2A.Z | downregulated | downregulated |
| P0CG49 | Polyubiquitin-B | downregulated | downregulated |
| P10126 | Elongation factor 1-alpha 1 | upregulated | - |
| P13405 | Retinoblastoma-associated protein | downregulated | downregulated |
| P14131 | 40S ribosomal protein S16 | downregulated | downregulated |
| P16627 | Heat shock 70 kDa protein 1-like | downregulated | - |
| P17156 | Heat shock-related 70 kDa protein 2 | upregulated | - |
| P18872 | Guanine nucleotide-binding protein G(o) subunit alpha | upregulated | - |
| P20029 | Endoplasmic reticulum chaperone BiP | upregulated | upregulated |
| P27661 | Histone H2AX | downregulated | downregulated |
| P32067 | Lupus La protein homolog | upregulated | upregulated |
| P42230 | Signal transducer and activator of transcription 5A | upregulated | - |
| P46737 | Lys-63-specific deubiquitinase BRCC36 | - | upregulated |
| P47962 | 60S ribosomal protein L5 | - | downregulated |
| P57780 | Alpha-actinin-4 | - | upregulated |
| P61982 | 14-3-3 protein gamma | downregulated | - |
| P62737 | Actin, aortic smooth muscle | upregulated | downregulated |
| P62843 | 40S ribosomal protein S15 | - | downregulated |
| P62908 | 40S ribosomal protein S3 | upregulated | - |
| P63085 | Mitogen-activated protein kinase 1 | downregulated | downregulated |
| P68040 | Receptor of activated protein C kinase 1 | upregulated | downregulated |
| P68254 | 14-3-3 protein theta | downregulated | - |
| P68368 | Tubulin alpha-4A chain | downregulated | - |
| P68372 | Tubulin beta-4B chain | downregulated | downregulated |
| P70290 | 55 kDa erythrocyte membrane protein | downregulated | downregulated |
| P84244 | Histone H3.3 | - | downregulated |
| P97813 | Phospholipase D2 | - | upregulated |
| P99024 | Tubulin beta-5 chain | downregulated | downregulated |
| Q01063 | cAMP-specific 3',5'-cyclic phosphodiesterase 4D | upregulated | - |
| Q01853 | Transitional endoplasmic reticulum ATPase | - | downregulated |
| Q02111 | Protein kinase C theta type | downregulated | downregulated |
| Q02248 | Catenin beta-1 | downregulated | upregulated |
| Q02566 | Myosin-6 | upregulated | downregulated |
| Q0VGT2 | Zinc finger protein GLI2 | downregulated | downregulated |
| Q5S006 | Leucine-rich repeat serine/threonine-protein kinase 2 | downregulated | downregulated |
| Q5SWU9 | Acetyl-CoA carboxylase 1 | - | upregulated |
| Q5SX39 | Myosin-4 | upregulated | downregulated |
| Q61188 | Histone-lysine N-methyltransferase EZH2 | upregulated | - |
| Q61696 | Heat shock 70 kDa protein 1A | upregulated | - |
| Q61879 | Myosin-10 | upregulated | - |
| Q63844 | Mitogen-activated protein kinase 3 | downregulated | downregulated |
| Q64511 | DNA topoisomerase 2-beta | - | downregulated |
| Q7TMM9 | Tubulin beta-2A chain | downregulated | - |
| Q7TPR4 | Alpha-actinin-1 | upregulated | upregulated |
| Q8BFZ3 | Beta-actin-like protein 2 | upregulated | downregulated |
| Q8BG05 | Heterogeneous nuclear ribonucleoprotein A3 | upregulated | upregulated |
| Q8BZ98 | Dynamin-3 | - | downregulated |
| Q8C2B3 | Histone deacetylase 7 | upregulated | upregulated |
| Q8K1M6 | Dynamin-1-like protein | - | upregulated |
| Q8VDW0 | ATP-dependent RNA helicase DDX39A | upregulated | upregulated |
| Q91VC3 | Eukaryotic initiation factor 4A-III | downregulated | - |
| Q91Y86 | Mitogen-activated protein kinase 8 | - | downregulated |
| Q91Z83 | Myosin-7 | upregulated | downregulated |
| Q922F4 | Tubulin beta-6 chain | downregulated | downregulated |
| Q99JY9 | Actin-related protein 3 | - | upregulated |
| Q99NB8 | Ubiquilin-4 | upregulated | - |
| Q9CWF2 | Tubulin beta-2B chain | downregulated | - |
| Q9D6F9 | Tubulin beta-4A chain | downregulated | downregulated |
| Q9D6P8 | Calmodulin-like protein 3 | - | upregulated |
| Q9ERD7 | Tubulin beta-3 chain | downregulated | downregulated |
| Q9ESX5 | H/ACA ribonucleoprotein complex subunit DKC1 | downregulated | downregulated |
| Q9JI91 | Alpha-actinin-2 | upregulated | - |
| Q9Z0R4 | Intersectin-1 | upregulated | downregulated |
| Q9Z1N5 | Spliceosome RNA helicase Ddx39b | upregulated | upregulated |
| Q9Z2V5 | Histone deacetylase 6 | upregulated | - |

^a^ The protein accession number was provided by the Uniprot database (http://www.uniprot.org/) ^b,c^ Protein status in comparison 10 mgF/L vs Control (T10xC) and 50 mgF/L vs Control (T50xC). The sign (-) indicates no difference in expression.
