# Supplementary figures and images for: Effects of long-term fluoride exposure are associated with oxidative biochemistry impairment and global proteomic modulation, but not genotoxicity, in parotid glands of mice
Source: PLoS One. 2022 Jan 27;17(1):e0261252. doi: 10.1371/journal.pone.0261252 (PMC8794182; doi:10.1371/journal.pone.0261252)

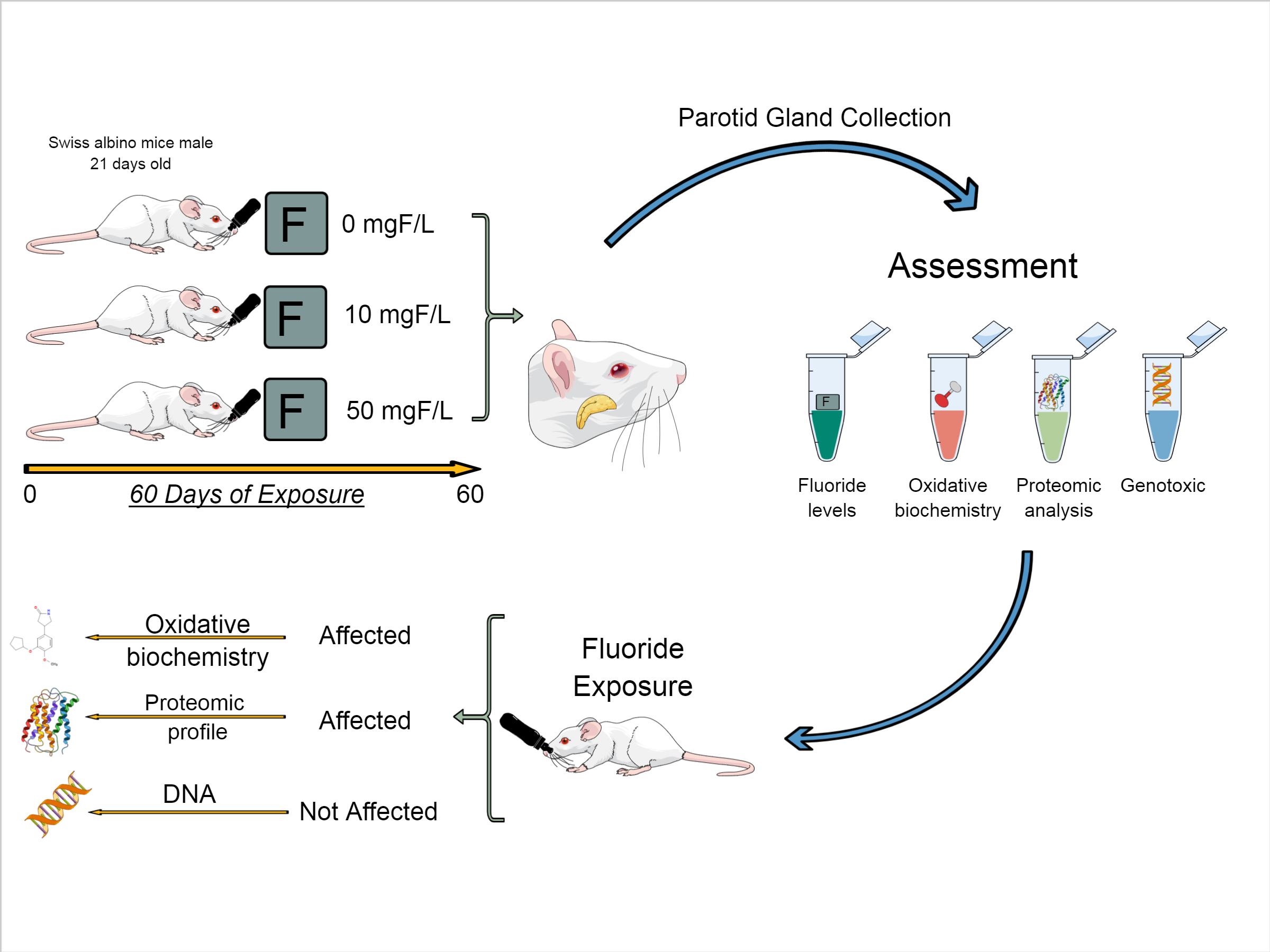

Supplement: S1 Graphical abstract — (TIF) [file pone.0261252.s004.tif]
